# Perovskite microcells fabricated using swelling-induced crack propagation for colored solar windows

**DOI:** 10.1038/s41467-022-29602-z

**Published:** 2022-04-11

**Authors:** Woongchan Lee, Young Jin Yoo, Jinhong Park, Joo Hwan Ko, Yeong Jae Kim, Huiwon Yun, Dong Hoe Kim, Young Min Song, Dae-Hyeong Kim

**Affiliations:** 1 Center for Nanoparticle Research, Institute for Basic Science (IBS), Seoul, 08826 Republic of Korea; 2 School of Chemical and Biological Engineering, Institute of Chemical Processes, Seoul National University, Seoul, 08826 Republic of Korea; 3 School of Electrical Engineering and Computer Science, Gwangju Institute of Science and Technology, Gwangju, 61005 Republic of Korea; 4 Department of Materials Science and Engineering, Korea University, Seoul, 02841 Republic of Korea; 5 Department of Materials Science and Engineering, Seoul National University, Seoul, 08826 Republic of Korea

**Keywords:** Solar cells, Surface patterning

## Abstract

Perovskite microcells have a great potential to be applied to diverse types of optoelectronic devices including light-emitting diodes, photodetectors, and solar cells. Although several perovskite fabrication methods have been researched, perovskite microcells without a significant efficiency drop during the patterning and fabrication process could not be developed yet. We herein report the fabrication of high-efficiency perovskite microcells using swelling-induced crack propagation and the application of the microcells to colored solar windows. The key procedure is a swelling-induced lift-off process that leads to patterned perovskite films with high-quality interfaces. Thus, a power conversion efficiency (PCE) of 20.1 % could be achieved with the perovskite microcell, which is nearly same as the PCE of our unpatterned perovskite photovoltaic device (PV). The semi-transparent PV based on microcells exhibited a light utilization efficiency of 4.67 and a color rendering index of 97.5 %. The metal–insulator–metal structure deposited on the semi-transparent PV enabled to fabricate solar windows with vivid colors and high color purity.

## Abstract (web summary)

Perovskite microcells can be applied to various types of optoelectronic devices. Here, authors report high efficiency perovskite microcells fabricated using swelling-induced crack propagation and demonstrate solar windows using the microcells.

## Introduction

Perovskite materials have been researched for the next-generation optoelectronics owing to their excellent properties, including the excellent photoabsorbance^1,2^, high carrier lifetime^3,4^, and tunable bandgap^5,6^. Recently, perovskite-patterning methods have been reported for the fabrication of microcells, which is essential for the perovskite optoelectronic devices that require array-type structures (e.g., light-emitting diodes^7^, microdisk lasers^8–10^, and photodetectors^11,12^). In addition, as perovskite solar cells recorded a certificated power-conversion efficiency (PCE) of 25.5%^13^, there have been attempts to fabricate semitransparent photovoltaic devices (PVs) using the perovskite microcell array^14–16^. Such semitransparent perovskite PVs can be also applied to colored solar windows installed in buildings instead of conventional windows.

Solar windows convert incident light to electricity and allow light transmittance like conventional windows. In particular, from an aesthetic point of view, colored types of solar windows have been highlighted. However, efforts to increase PCEs usually decrease average visible transmittances (AVTs) and color-rendering indices (CRIs). Especially for colored types, PCEs are dependent on their designed color^17,18^. Thus, light-utilization efficiency (LUE, calculated by AVT × PCE), which represents characteristics of windows and solar cells at the same time, is used as a key parameter in semitransparent PVs^19,20^. Also, CRI, a figure of merit for the transmitted color, can quantitatively measure how accurately the colors are rendered^21^. Since the AVT and average power draw per unit area of the typical tinted windows are > 50% and 5 mW/cm^2^, respectively, semitransparent PVs with a LUE of >2.5 and with an AVT of >50% can be considered as ideal candidates for solar windows^19,22^. Besides, a CRI of >90% is required for the high-quality window in the window industry^23,24^. It is also important that the PCE of colored solar windows should be constant, regardless of color.

To fabricate the high-performance perovskite microcell, devising a sophisticated patterning process is essential since it determines the quality of the patterned perovskite film such as the shape, uniformity, and crystallinity, as well as the performance of the microcell^7–12,16,25–27^. Numerous patterning methods, such as electrochemical anodization^28,29^, nanoimprint^30^, and focused ion-beam etching^31^, have been reported for the fabrication of perovskite microcells. Among them, a dewetting-based patterning method^12,16^, which can deposit a perovskite layer on selected regions by controlling the surface energy of a substrate, has been widely studied. However, deformed shapes and voids are often observed in the patterned perovskite films, which decrease the device performance. Thus, a new patterning method has been requested for high-performance perovskite microcells.

Recently, a lift-off-based patterning method using poor adhesion polymers such as parylene has been reported^32^. In this case, a perovskite film can be uniformly deposited since the surface energy is uniform throughout the entire prepatterned substrate. However, when removing the poor adhesion polymer from the substrate, a large stress is induced on the entire film, which can cause fractures or partial delamination of patterns^32^. In particular, these fractures or partial delamination of patterns can easily occur in high-efficiency perovskite PVs since perovskite films for high-efficiency PVs are usually formed through the surface passivation by adding excess lead iodide (PbI_2_)^33^ or two-dimensional (2D) perovskite materials^34^, which improves the properties but lowers the fracture energy of the perovskite films^35,36^. Therefore, a novel patterning method is required to fabricate uniform perovskite patterns for high-efficiency microcells that can lead to the high-performance colored solar windows.

Here, we present a novel lift-off-based patterning method using swelling-induced crack propagation to fabricate high-efficiency perovskite microcells. The swelling-induced lift-off method allows the fabrication of a flat, uniform, crystalline, and patterned perovskite film without defects such as fracture or partial delamination. In addition, the simultaneous lift-off patterning of the perovskite layer and electron- transport layer (ETL) minimizes interfacial defects. The high-quality patterned film and interface enable to fabricate a microcell with a PCE of 20.1%, nearly the same as the PCE of our unpatterned perovskite PV. Through optimization of the microcell size and cell-to-cell distance, as well as adoption of multifunctional moth-eye nanostructures^37–40^, the semitransparent perovskite PV exhibits a LUE of 4.67 and a CRI of 97.5%. These performances represent distinctive LUEs and CRIs among previously reported semitransparent PVs (c.f., PCEs, AVTs, CRIs, LUEs, and LUE × CRIs are plotted in Supplementary Figs. 1, 2). Besides, the incorporation of a metal–insulator–metal (MIM) resonant structure on the semitransparent PV enables to fabricate a colored solar window with high color purity, leading to nearly constant PCEs, regardless of color (c.f., PCE × saturations, AVTs, and LUE × saturations are plotted in Supplementary Fig. 3)^41^.

## Results

### Swelling-induced lift-off method for high-quality patterned perovskite films

Patterning vapor-deposited materials by using the lift-off method can lead to desired pattern shapes without fractures or partial delamination because the vapor-deposited film on the patterned sacrificial layer is typically not connected to the film on the bottom substrate (Supplementary Fig. 4a)^42^. However, since perovskite materials are usually solution-processed, the perovskite film on the patterned sacrificial layer and that on the bottom substrate are connected to each other at the pattern edge (Supplementary Fig. 4b). When it comes to the lift-off pattering of the solution-processed perovskite film, thereby, cracks are generated and propagate randomly, and the fracture and/or partial delamination of the patterned films occur. Therefore, the control of the crack generation and propagation along the edge of the pattern is important for the lift-off patterning of a perovskite film without fractures or partial delamination. Thus, we used swelling-induced crack propagation for the lift-off patterning of the solution-processed perovskite film.

**Fig. 1:**
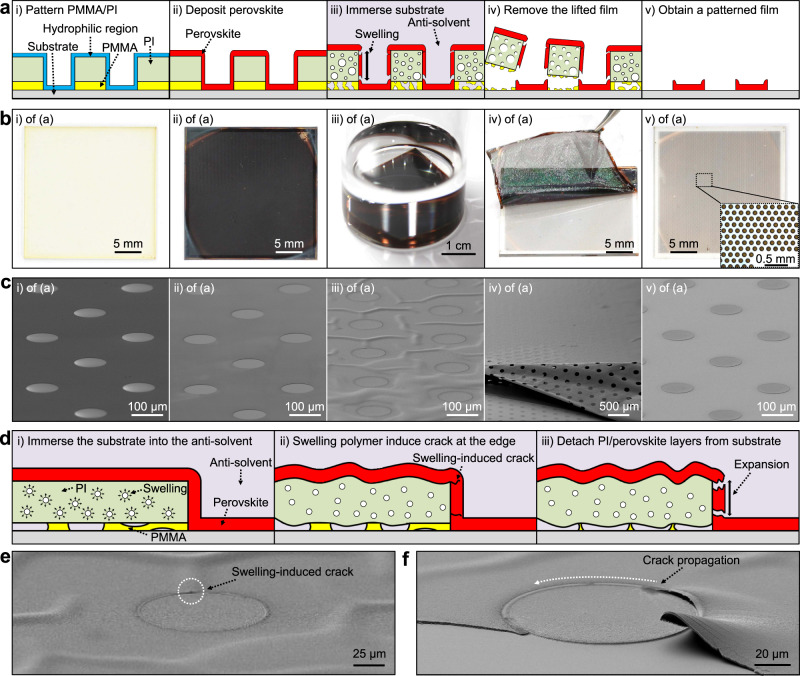
Swelling-induced lift-off method for high-quality perovskite films. **a** Cross-sectional schematic illustrations of the swelling-induced lift-off method for the step-by-step procedure. **b** Optical camera images and **c**, scanning electron microscopy (SEM) images that correspond to frames of (**a**). The inset shows an optical microscopy image of the patterned perovskite film. **d** Schematic illustrations that explain the crack-generation procedure in (iii) of Fig. 1a. **e**, **f** SEM images that show the generated crack (**e**) and its propagation (**f**).

Figure 1a shows cross-sectional schematic illustrations, which account for step-by-step procedures of the swelling-induced lift-off method for the fabrication of a perovskite dot array. Optical camera and scanning electron microscopy (SEM) images, which correspond to frames of Fig. 1a, are shown in Fig. 1b, c, respectively. A substrate (gray) spin-coated with poly(methyl methacrylate) (PMMA) and polyimide (PI) is prepared. Then, PMMA (yellow) and PI (green) are patterned via the oxygen plasma etching, which modifies the surface hydrophilic (i of Fig. 1a–c). In this process, the top SiO_2_ layer plays a role as an etch stop layer for the dry etching of PMMA/PI layers and is finally wet-etched using a buffered oxide etchant (BOE). Next, a perovskite film (red) is deposited by spin-coating on the prepatterned PMMA/PI layers (ii of Fig. 1a–c). The entire substrate is immersed into an antisolvent (e.g., chloroform, pale purple) (iii of Fig. 1a–c), which dissolves PMMA and makes PI swell (i, ii of Fig. 1d). The slowly swollen PI layer applies mechanical stress on the edge of the perovskite dot pattern and forms cracks at the pattern edge (iii of Fig. 1d, e). After cracks fully propagate along the edge (Fig. 1f), the PI/perovskite layers are easily detached from the substrate without any fractures or partial delamination of the patterned films on the substrate (iv, v of Fig. 1a–c). Supplementary Movie 1 shows the detailed peeling-off procedure.

Orthogonality of the antisolvent to the perovskite layer preserves the quality of the original film. According to X-ray diffraction (XRD) analysis, the patterned perovskite film has the same ratio of FAMAPbI_3-x_Br_x_ and PbI_2_ as that of the original film (Supplementary Fig. 5). Moreover, δ-phase FAPbI_3_, which may degrade the performance of PVs, is not detected in the XRD result^43^.

### Perovskite patterns fabricated by the swelling-induced lift-off method and dewetting method

**Fig. 2:**
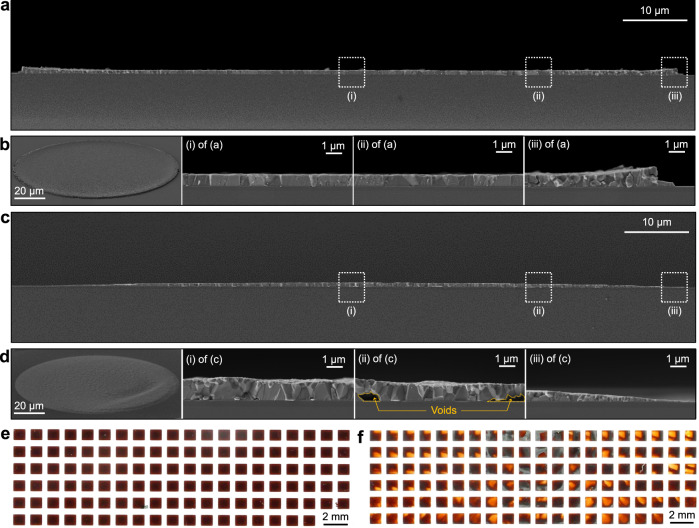
Perovskite patterns fabricated by the swelling-induced lift-off method and the dewetting method. **a** Cross-sectional SEM image of a perovskite dot pattern fabricated by the swelling-induced lift-off method. **b** Tilted view (left frame) and magnified views (other frames) of Fig. 2a. **c** Cross-sectional SEM image of a perovskite dot pattern fabricated by the dewetting method. **d** Tilted view (left frame) and magnified views (other frames) of Fig. 2c. **e**, **f** Optical camera images that show the array of rectangular perovskite patterns fabricated by the swelling-induced lift-off method (**e**) and the dewetting method (**f**).

During the prepatterning of the PMMA/PI layers, the substrate becomes hydrophilic owing to oxygen plasma (i of Supplementary Fig. 6a). Thus, the perovskite precursor can be spread over the entire substrate (ii, iii of Supplementary Fig. 6a). This enables the deposition of a perovskite film with a uniform thickness, a flat surface, and conformal coverage (iv of Supplementary Fig. 6a). The cross-sectional and tilted-view image of the perovskite dot pattern are shown in Fig. 2a and in the left frame of Fig. 2b, respectively. Additional images for the magnified cross-sectional view at the center (i of Fig. 2a), off the center (ii of Fig. 2a), and near the edge (iii of Fig. 2a) are shown in other frames of Fig. 2b.

Such a highly uniform and void-free patterned perovskite film cannot be fabricated with previously reported patterning methods. In the case of the dewetting-based patterning method (dewetting method)^12^, for example, the spin-coated precursor staying on hydrophilic regions of the substrate shows deformed shapes due to the centrifugal force during the spin-coating, which leads to a nonuniform pattern of the perovskite film (Supplementary Fig. 6b). The cross-sectional, tilted, and magnified views of the patterned perovskite film by the dewetting method show nonuniform thickness, voids, and high surface roughness (Fig. 2c, d). In addition, the insufficient volume of the perovskite precursor at the edges results in the defective and deficient perovskite crystal growth at the edges of the patterned perovskite film (Fig. 2d)^12^.

Large-scale perovskite films are patterned as an array of rectangles by the swelling-induced lift-off method and dewetting method for further comparison. The swelling-induced lift-off method results in a uniformly patterned array of rectangular perovskite films (Fig. 2e). But, the array of rectangular perovskite films patterned by the dewetting method shows poor cell-to-cell uniformity (Fig. 2f).

### High-performance perovskite microcells with reduced interfacial defects

**Fig. 3:**
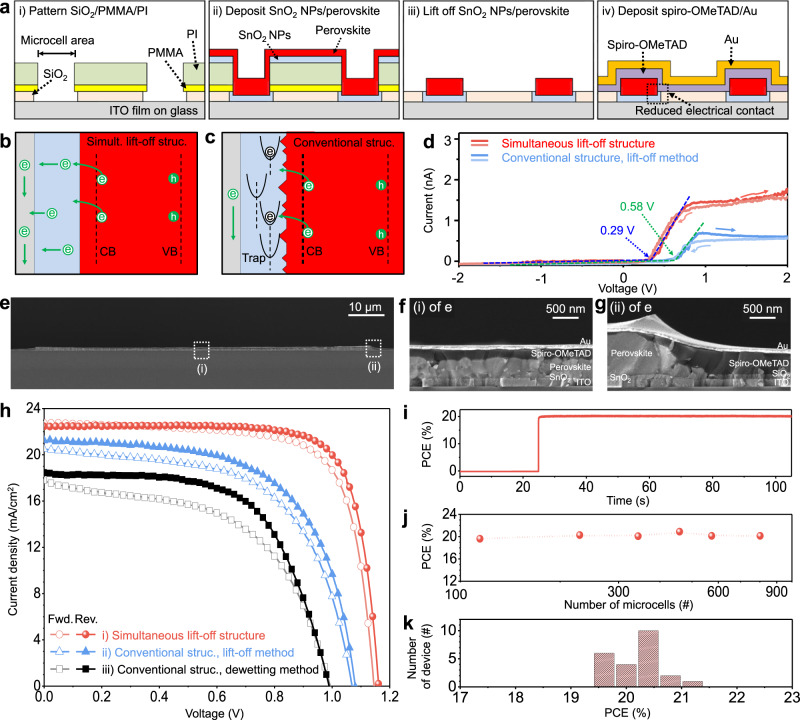
Perovskite microcells with reduced interfacial defects. **a** Cross-sectional illustrations of the fabrication process of perovskite microcells. **b**, **c** Schematic illustrations of the charge-carrier dynamics in the perovskite microcell with the simultaneous lift-off structure (**b**) and the conventional structure (**c**). **d** Current–voltage (*I–V*) characteristics of the ETL/perovskite layer with the simultaneous lift-off structure (red) and the conventional structure (blue) in the dark condition. Pointed values and arrows indicate threshold voltages and scan directions, respectively. **e** Cross-sectional SEM image of a perovskite microcell. **f**, **g** Magnified view of Fig. 3e at the center (**f**) and at the edge (**g**). **h** Current density–voltage (*J–V*) characteristics of three types of patterned devices under the forward and reverse scan. **i** Stabilized power output of perovskite microcells. **j** PCEs (reverse scan) depending on the number of perovskite microcells in the PV. **k** Statistical data on the PCE of 25 PVs (reverse scan).

The formation of a high-quality interface between charge-transport layers (CTLs) and perovskite layers plays an important role to fabricate high-performance perovskite PVs. Figure 3a illustrates the fabrication process of perovskite microcells with excellent interfaces by utilizing and modifying the swelling-induced lift-off method. First, PMMA (yellow), PI (green), and SiO_2_ (beige) layers are patterned on the indium-doped tin oxide (ITO, gray)/glass substrate. Photolithography and dry etching using O_2_ plasma are applied to PMMA and PI, and wet etching using a BOE solution is applied to SiO_2_. The patterned SiO_2_ serves as an insulation layer between the ITO electrode and the hole-transport layer (HTL) (i of Fig. 3a). Then, an ETL (SnO_2_ nanoparticles (NPs), blue) and a perovskite layer (red) are spin-coated (ii of Fig. 3a). These two films are simultaneously patterned by the swelling-induced lift-off method. The patterned ETL/perovskite layers, isolated by SiO_2_, remain on the ITO electrode (iii of Fig. 3a). Finally, a HTL (spiro-OMeTAD, violet) and a top electrode (Au, orange) are deposited (iv of Fig. 3a). Due to the simultaneous patterning of the ETL and perovskite layer, generation of additional interfacial defects can be significantly suppressed (Fig. 3b). This device structure with reduced interfacial defects is called as a “simultaneous lift-off structure” (Supplementary Fig. 7a)^32^.

For further analysis of the device performance by using the simultaneous lift-off structure, we also fabricated a perovskite microcell with a conventional structure for comparison. In the conventional fabrication process for patterned cells (Supplementary Fig. 7b), since etching of the insulation layer is needed after the ETL is deposited on the bottom electrode, the surface condition of the ETL is not ideal due to the etching damage, which leads to an efficiency drop^16^. Specifically, an insulation layer (beige) is deposited on the ETL (blue) to prevent a direct contact between the ETL and the HTL (violet)^16^. Then, the insulation layer is patterned and etched to expose the ETL inside the microcell area. Afterward, the perovskite layer (red) is deposited and patterned, followed by the deposition of the HTL and top electrode. Etching of the insulation layer damages the ETL surface, and thereby defects are generated at the interface between the ETL and the perovskite layer, which induces the delayed transfer of electrons (Fig. 3c)^44^. This device structure with interfacial defects is called as a “conventional structure”.

Atomic force microscopy (AFM) and conductive AFM are used to investigate the different surface properties of the patterned ETL/perovskite layers between the simultaneous lift-off structure and the conventional structure. Although morphological differences are negligible (Supplementary Fig. 8a), the surface conductivity and the number of conductive regions of the ETL/perovskite layer with the simultaneous lift-off structure are higher than those of the ETL/perovskite layer with the conventional structure (Supplementary Fig. 8b). This difference can be confirmed by the higher threshold voltage and internal resistance of the perovskite microcell with the conventional structure than those of the perovskite microcell with the simultaneous lift-off structure (Fig. 3d)^45^.

Figure 3e–g and Supplementary Fig. 9 show cross-sectional SEM images and transmission electron microcopy (TEM) images with the energy-dispersive spectroscopy (EDS) analysis of the perovskite microcell with the simultaneous lift-off structure, respectively. The perovskite microcells have the same dot-array pattern in Fig. 1. The SEM confirms the flat and uniform thickness of the layers at the center of the perovskite microcell. The configuration of device layers at the center of the perovskite microcell (i.e., ITO/SnO_2_ NPs/perovskite layer/spiro-OMeTAD/Au) is shown in Fig. 3f. The edge structure of the perovskite microcell is shown in Fig. 3g and Supplementary Fig. 9. Note that etching conditions of the bottom SiO_2_ affect the edge structure of the perovskite microcell. Dry etching via O_2_ plasma makes the PMMA/PI multilayers hydrophilic and enables the hydrophilic SnO_2_ NPs to be deposited on the sidewall of PMMA/PI multilayers (Supplementary Fig. 10a). Unlike the dry etching, however, since the wet etching using a buffered oxide etchant cannot modify the surface energy of the PMMA/PI multilayers, the deposition of the SnO_2_ NP solution on the sidewall is suppressed due to the high surface energy of the PMMA/PI multilayers (Supplementary Fig. 10b). Also, the bottom SiO_2_ layer is deliberately undercut during the wet-etching process to prevent the deposition of the ETL on the sidewall, which minimizes the contact between the ETL and HTL (Fig. 3g and Supplementary Figs. 9, 10).

The high-quality perovskite layer and interface enable to fabricate high-performance perovskite microcells. The perovskite microcell with the simultaneous lift-off structure exhibits a PCE of 20.1% with an open-circuit voltage (*V*_OC_) of 1.16 V, a short-circuit current density (*J*_SC_) of 22.5 mA/cm^2^, and a fill factor (FF) of 77% under AM 1.5 illumination, which are almost the same as those of our unpatterned perovskite PV (Supplementary Fig. 11a). In total, 458 perovskite microcells with a diameter of 100 μm (Supplementary Fig. 11b) and an electrode area of 9 mm^2^ were used for the measurement (scan rate of 100 mV/s, without UV filter).

To compare the effect of the device structure and patterning method, we fabricated three kinds of devices: (i) device with the simultaneous lift-off structure, (ii) device with the conventional structure fabricated by the lift-off method, and (iii) device with the conventional structure fabricated by the dewetting method (Fig. 3h). The increased threshold voltage and internal resistance due to the etching damage deteriorate performance of the device with the conventional structure. The deformed shapes and voids of the perovskite film fabricated by the dewetting method lead to worse performance. The stabilized power output (SPO) of the perovskite microcell with the simultaneous lift-off structure is 19.9% (bias ~ 0.95 V, Fig. 3i). PCEs are maintained, while the number of perovskite microcells with the simultaneous lift-off structure is increased from 114 to 802 in a fixed area of 9 mm^2^ (Fig. 3j). A histogram of PCEs shows statistical data of 25 PVs, which shows reproducibility (Fig. 3k). The external quantum efficiency (EQE) and integrated *J*_*sc*_ of the perovskite microcell with the simultaneous lift-off structure are shown in Supplementary Fig. 12. The integrated *J*_*sc*_ calculated from EQE is 21.76 mA/cm^2^, which is well matched (within 4%) to the measured *J*_*sc*_ under the solar simulator.

### Semitransparent photovoltaic device based on perovskite microcells

**Fig. 4:**
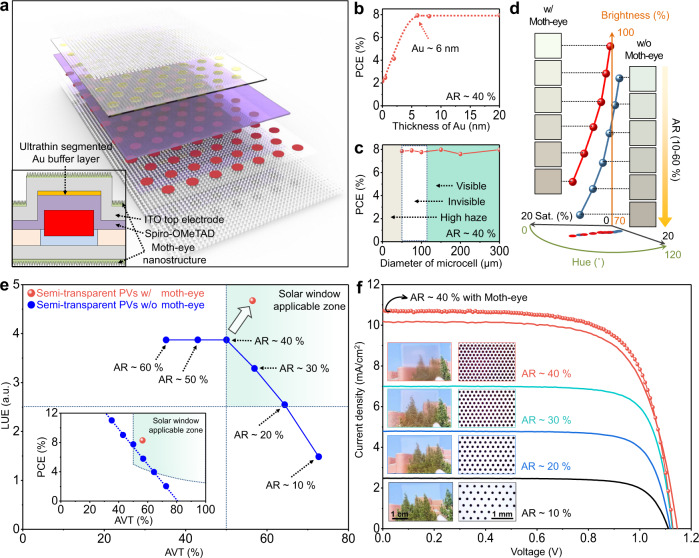
Semitransparent PV with an ultrathin segmented Au buffer layer and moth-eye-inspired nanostructures. **a** Schematic exploded view of the semitransparent PV. The inset shows a magnified cross-sectional view of the perovskite microcell in the semitransparent PV. **b** PCEs (reverse scan) of semitransparent PVs, depending on the thickness of the segmented Au buffer layer. The area ratio (AR) is fixed at 40%. **c** PCEs (reverse scan) of semitransparent PVs, depending on the diameter of the microcell. The AR is fixed at 40%. **d** Color representation and HSB color map of the semitransparent PV with and without the moth-eye nanostructures for various ARs (10−60%). **e** Light-utilization efficiencies (LUEs) of semitransparent PVs with and without moth-eye nanostructures (red and blue circle, respectively) for various AVTs. The inset shows PCEs in Fig. 4e for various AVTs. PCEs obtained from the forward and reverse scan are averaged. The green zones in Fig. 4e and its inset represent the AVT and LUE criteria suitable for solar windows (AVT and LUE need to be over 50% and 2.5, respectively). **f** *J*–*V* characteristics of semitransparent PVs without (solid line) and with (dotted line) the moth-eye nanostructures (reverse scan). The inset shows optical camera (left) and microscopy (right) images of semitransparent PVs, depending on the AR.

Figure 4a shows an exploded illustration of the semitransparent PV. A dot-array pattern based on perovskite microcells with the simultaneous lift-off structure is adopted for the semitransparent PV. The inset of Fig. 4a shows a cross-sectional illustration of a single microcell. An ITO electrode is applied to the top electrode to enhance light transmittance. As a bifunctional layer, for the enhanced charge transport and the protection from energetic damages during ITO sputtering, an ultrathin and segmented Au buffer layer (patterned inside the microcell area) is inserted between the HTL and the top ITO electrode (Supplementary Fig. 13a)^46^. Since the PCE is saturated with the thickness of segmented Au buffer layer above 6 nm (Fig. 4b and Supplementary Fig. 13b), its thickness was set as 6 nm for high transmittance. The perovskite microcells with the ultrathin segmented Au buffer layer (inside microcell area) and ITO top electrode (entire device area) exhibit higher AVTs than the perovskite microcells with the ultrathin Au top electrode (6 nm, entire device area) (Supplementary Fig. 14a), resulting in higher LUEs (Supplementary Fig. 14b). The transmittance spectra, CIE coordinate, and haze of semitransparent PVs are shown in Supplementary Fig. 15 and Supplementary Table 3.

The human eye has an angular resolution of ~1 arcminute^47^, which corresponds to ~120 μm at a distance of ~40 cm. Thus, perovskite microcells cannot be perceived with bare eyes when the cell diameter is <120 μm (Supplementary Fig. 16). However, when the diameter is <50 μm, haze significantly increases owing to light scattering (Supplementary Fig. 17). Thus, a microcell diameter of 100 μm is used for the semitransparent PV. Meanwhile, semitransparent PVs maintain stable PCEs, regardless of the diameter of perovskite microcells between 50 μm and 300 μm under a fixed ratio of the microcell area to the entire device area (Fig. 4c).

To maximize transparency and enhance absorption, moth-eye nanostructures are added on top and bottom of the semitransparent PV. Moth-eye nanostructures (Supplementary Fig. 18a, inset) made of soft and hard polydimethylsiloxane (s-PDMS and h-PDMS) with a gradual refractive index profile (Supplementary Fig. 18b), are transferred to both sides of the semitransparent PV (Fig. 4a inset)^48^. The nanostructures improve transmittance (Supplementary Fig. 18c) and chromatic properties (e.g., hue, saturation, and brightness (HSB)) (Fig. 4d), because the nanostructure with a sub-wavelength-scale dimension guides visible light. It also enhances light absorption over a wide range of incident angles (Supplementary Fig. 18d)^49^.

Figure 4e and its inset show LUEs and PCEs of semitransparent PVs with (red) and without (blue) moth-eye nanostructures. LUEs reach 4.67 (AVT ~56.45%, PCE ~8.28%, and CRI ~97.5% for AR ~40%) with the nanostructure and 3.87 (AVT ~50.04%, PCE ~7.74%, and CRI ~97.2% for AR ~40%) without the nanostructure (Supplementary Figs. 1, 2 and Supplementary Table 1). The current density–voltage (*J–V)* characteristics and photovoltaic parameters (for AVTs ≥50% in Fig. 4e) are shown in Fig. 4f and Supplementary Table 2, respectively. Figure 4f insets show optical camera and microscopy images of semitransparent PVs with various area ratios (ARs) (microcell diameter: 100 μm). The PCE increases as the AR increases (Supplementary Fig. 19a), and statistical data show reproducibility (Supplementary Fig. 19b).

### Colored solar window using the semitransparent photovoltaic device

**Fig. 5:**
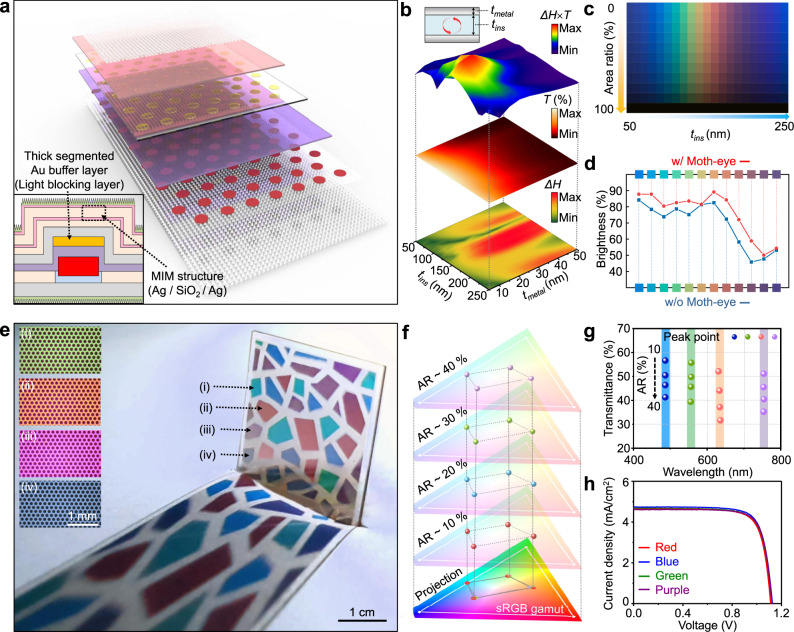
Colored solar window with metal–insulator–metal structures and moth-eye-inspired nanostructures. **a** Schematic exploded view of the colored solar window with the metal–insulator–metal (MIM) resonant structure. The inset shows a cross-sectional view of the perovskite microcell in the colored solar window. **b** Contour plots of color difference (Δ*H*), transmittance (*T*), and product of two values (Δ*H* × *T*) for optimization of the thicknesses of insulator (*t*_ins_) and metal (*t*_metal_). The upper-left inset shows a schematic illustration of the MIM structure. **c** Color palette for various ARs and *t*_ins_. **d** Chromatic brightness of the colored solar window with and without the moth-eye nanostructures. **e** Photograph of a mosaic-patterned colored solar window with four different colors (i.e., red, green, blue, and purple). The inset shows optical microscopy images of four PVs ((i) green, (ii) red, (iii) purple, and (iv) blue). **f** Chromaticity values on the CIE coordinate for various ARs (10–40%). **g** Transmittance values at peak points for colored solar windows with various ARs (10–40%). **h** *J*–*V* characteristics (reverse scan) of colored solar windows with various colors (red, blue, green, and purple).

Figure 5a shows an exploded illustration of the colored solar window. The basic structure is identical to that of the semitransparent PV, except for additional MIM resonant structures for expressing color^50,51^ and a thick segmented Au buffer layer (70 nm) under the top ITO electrode for high chromaticity. The inset of Fig. 5a shows a cross-sectional view of the perovskite microcell. Owing to light resonance inside the structure (Supplementary Fig. 20a), the MIM structure allows light transmission only at the specific wavelength, which is determined by the insulator thickness. By suppressing surface reflection (Supplementary Fig. 20b), moth-eye nanostructures enhance transmittance up to ~10% (Supplementary Fig. 21).

Color difference (Δ*H*) and transmittance (*T*) of the MIM structure depend on the thickness of metal and insulator. The optimal design for high color purity and transmittance is found at the highest value of Δ*H* × *T*, which corresponds to a metal thickness of ~15 nm (Fig. 5b). The color varies depending on the insulator thickness (horizontal axis of Fig. 5c). As the AR increases, each color expressed by the MIM structure is maintained, but brightness decreases due to reduced light transmission (vertical axis of Fig. 5c). Moth-eye nanostructures improve brightness according to HSB chromaticity calculations (Fig. 5d)^52^. Chromatic saturation is a degree of color purity in each hue, which is important in colored window applications since it affects the whitish shift of the transmissive light’s color^19^. LUE × saturation of the colored solar windows reaches 116.8 with stable PCEs for different colors (i.e., red, green, and blue), higher than those from previous reports (Supplementary Fig. 3 and Supplementary Table 4).

A colored solar window with a mosaic pattern of four colors (i.e., red, green, blue, and purple) is shown in Fig. 5e. Transmissive light through the PV is projected onto a white floor. By controlling the insulator thickness in the MIM structure, various colors can be displayed (Fig. 5e inset). The background is seen through the colored solar windows with various colors (Supplementary Fig. 22a). According to transmittance-measurement data in the visible-light range, the position of the peak wavelength in the transmittance spectra does not change for different ARs (Fig. 5f, g and Supplementary Fig. 22b–d), but the AVT of the colored solar window is lower than that of the semitransparent PV due to the thick segmented Au buffer layer (Supplementary Fig. 22e). Nearly the same chromaticity is maintained for different ARs (Fig. 5g) owing to the thick segmented Au buffer layer, which cuts off transmittance in the wavelength range over 600 nm (Supplementary Fig. 23a). With an ultrathin Au buffer layer (6 nm), light with the wavelength over 600 nm is also transmitted (Supplementary Fig. 23b), and thus the chromaticity changes according to the AR (Supplementary Fig. 23c). Since the MIM structure is applied to the backside of the semitransparent PV and only works through the non-microcell area, the electrical properties of colored solar windows are nearly the same, regardless of color (Supplementary Fig. 24 and Fig. 5h).

## Discussion

We developed a swelling-induced lift-off method and a related fabrication process for perovskite microcells. The swollen PI formed cracks on the pattern edge of the perovskite layer and guided their propagation for the lift-off patterning of the perovskite film. The patterned perovskite film features the flat surface, uniform thickness, and high crystallinity without fractures or partial delamination. In addition, interfacial defects between the perovskite layer and CTLs could be suppressed owing to the simultaneous patterning process. These patterning methods and device structure have the great advantages in the fabrication of high-quality microcell arrays that can be applied to photovoltaic devices as well as photodetectors and light-emitting diodes (LEDs). By using this patterning method, we demonstrated the semitransparent perovskite PVs whose LUEs and CRIs were distinctive. The optimized moth-eye structure coated on both sides of the semitransparent perovskite PVs further enhances the optical properties of the devices. For the colored solar window, a MIM resonant structure with an optimized design for each color pattern was added to the semitransparent PV. The fabricated colored solar windows exhibit high chromaticity and constant PCE, regardless of color. These high-performance perovskite microcells are expected to become a big step toward the next-generation perovskite optoelectronics.

## Methods

### Materials

All materials were used as purchased, unless stated. The indium-doped tin oxide (ITO) glass (10 Ω/cm^2^, 0.7-mm thick, transmittance >85%) was purchased from AMG Tech (Korea). Formamidinium iodide (FAI), methylammonium iodide (MAI), methylammonium bromide (MABr), and methylammonium chloride (MACl) were purchased from Greatcell Energy (Australia). Poly(pyromellitic dianhydride-*co*-4,4′-oxydianiline), bis(trifluoromethane)-sulfonimide lithium salt (LiTFSI) (>99.95%), acetonitrile, chlorobenzene, and 4-*tert*-butylpyridine (96%) were purchased from Sigma Aldrich (USA). Tin oxide, ethanol, isopropanol, dimethylformamide (DMF), dimethylsurfoxide (DMSO), lead iodide (99.999%), and lead bromide (99.999%) were purchased from Alfa Aesar (USA). Methylamine (~40.0–43.0%) was purchased from Tokyo Chemical Industry (Japan), and spiro-OMeTAD (>99.9%) was purchased from Lumtec (Taiwan). Polydimethylsiloxane (PDMS) was purchased from Dow Corning Corporation (USA). Polymethyl methacrylate (PMMA) was purchased from Kayaku Advanced Materials (USA).

### Preparation of a prepatterned substrate for swelling-induced lift-off method

The patterned ITO glass was sequentially cleaned with deionized water, ethanol, acetone, and chloroform. Afterward, SiO_2_, PMMA, polyimide (PI), and SiO_2_ were deposited on the ITO glass in sequential order. Specifically, a SiO_2_ layer of 50 nm was deposited by sputtering at a rate of ~ 2 Å/s. PMMA was spin-coated with 3000 rpm for 30 s, and was annealed at 150 °C for 30 min in air. The PI precursor was spin-coated at 8000 rpm for 60 s, and was annealed at 250 °C for 3 h in air. Another SiO_2_ layer of 180 nm, which plays a role as an etch-stop layer for PI and PMMA, was deposited by sputtering at a rate of ~2 Å/s. After the deposition is finished, the multilayer (i.e., SiO_2_/PMMA/PI/SiO_2_) on the ITO glass was patterned via photolithography, wet etching using a BOE solution (~10 s), dry etching using O_2_ plasma (100 sccm of O_2_ gas, 150 W, 0.1 Torr, 4 min), and wet etching using a BOE solution (~8 s), subsequently. When the bottom SiO_2_ layer was patterned via wet etching, the top SiO_2_ layer was removed at the same time (Figs. 1a, 3a). In order to keep the spacing between adjacent patterns constant, the multilayer was patterned as a dot array with the hexagonal lattice.

### Fabrication of semitransparent PVs and colored solar windows

The diluted SnO_2_ nanoparticle (NP) solution (2.2% NPs in ethanol and water) was spin-coated on the prepatterned substrate with 6,500 rpm for 60 s and then annealed at 140 °C for 20 min in air. A solution of 1.1 M PbI_2_, 0.2 M PbBr_2_, 0.3 M MAI, and 0.1 M MABr in DMF/DMSO (4:1) was spin-coated with 2000 rpm for 8 s and 6500 rpm for 22 s. At the last 10 s of the spin-coating step, chlorobenzene was dropped on the spinning substrate. Subsequently, the sample was annealed at 70 °C for 1 min. A solution of 0.3 M FAI, 0.05 M MABr, and 0.07 M MACl in isopropanol was dropped on the sample and spun with 5000 rpm for 20 s. Finally, the sample was annealed at 130 °C for 20 min. The sample was immersed into the chloroform to dissolve the PMMA layer. After crack generation and propagation along the edge of the pattern, PI/perovskite layers were lifted off. Then, a solution of 85 mg of spiro-OMeTAD, a solution of 17.5 μl of LiTFSI (524 mg of LiTFSI dissolved in 1 mL of acetonitrile solvent), and 28.5 μl of tert-butylpyridine in 1 mL of chlorobenzene was dropped on the sample and spin-coated with 3000 rpm for 30 s. For the fabrication of the semitransparent PV, an ultrathin segmented Au buffer layer (6 nm) was deposited via thermal evaporation under high vacuum (~10^–6^ Torr) to protect the HTL during the sputtering of the top ITO electrode. To deposit the ultrathin segmented Au buffer layer only inside the microcell area, a shadow mask was used. The shadow mask was aligned by using an optical microscope and firmly fixed by magnets. Note that the misalignment of the shadow mask can cause the HTL to be exposed to the energetic ion bombardment damages, resulting in reduction of the PCE of the perovskite microcells. The ultrathin segmented Au buffer layer was deposited at a rate of ~0.5 Å/s up to a thickness of 6 nm. Then, the shadow mask was changed for an electrode area of 9 mm^2^, and a top ITO electrode (150 nm) was deposited at a rate of ~0.7 Å/s by using a sputter (Scientech, Korea) under high vacuum (~10^–6^ Torr). Instead of the ultrathin segmented Au buffer layer (6 nm) in the semitransparent PV, a thick segmented Au buffer layer (70 nm) was deposited inside the microcell area through the shadow mask to fabricate the colored solar window. The metal–insulator–metal structure was also deposited on the device to express various colors. An additional Au layer of 70 nm was deposited to fabricate contact pads for external wiring. Finally, moth-eye-inspired nanostructures were transferred onto both sides of semitransparent PVs and colored solar windows.

### Fabrication of an artificial moth-eye structure

A nickel mold for the artificial moth-eye structure was coated with a release agent for the facile peel-off of the polymer layer. The base polymer and the curing agent of h-PDMS and s-PDMS were mixed with a ratio of 1:1 and 10:1. The PDMS mixtures were placed in a vacuum chamber for 30 min for removing trapped air inside the polymer. First, the h-PDMS mixture was spin-coated for 30 s with 500 rpm on the mold. The h-PDMS layer was cured at 150 °C for 10 min on the hot plate. Then, the s-PDMS mixture was spin-coated with 300 rpm for 30 s. The s-PDMS layer was cured at 150 °C on the hot plate for 5 min and at room temperature for 30 min. Finally, the nanostructure made of PDMS was peeled off from the mold.

### Fabrication of metal–insulator–metal structures

Both metal (Ag) and insulator (SiO_2_) of the MIM structure were deposited by using an electron-beam evaporator (KVE-E2000, Korea Vacuum Tech Co., Korea) under high vacuum (~10^–6^ Torr). The Ag film was deposited at a rate of ~1 Å/s to a thickness of 15 nm. The SiO_2_ layer was deposited at a rate of ~0.5 Å/s to the target thickness.

### Characterization of the perovskite film

The surface morphology of the perovskite film was characterized by using a field-emission scanning electron microscope (FE-SEM, MERLIN Compact, ZEISS, Germany) with an in-lens detector in secondary electrons type-1 and -2 modes. The cross-sectional structure of the perovskite film and that of the photovoltaic device were observed by using a FE-SEM (MERLIN Compact, ZEISS, Germany). Transmission electron microscopy (TEM, JEM-2100F, JEOL, Japan) observation was performed at 200 kV. Energy-dispersive spectroscopy (EDS, X-MAX 80 T, Oxford, UK) was operated at 200 kV. X-ray diffraction (XRD) patterns were measured with a Bruker D8 diffractometer using Cu Kα radiation (*λ* = 1.54 Å).

### Device characterization

All device measurements were carried out at 20 °C under a humidity condition of 25%. Simulated AM 1.5 G irradiation (100 mWcm^−2^) was applied by a solar simulator (Newport, USA) with a Xenon Arc lamp 6255 (Newport, USA) for measuring current density–voltage (*J–V*) characteristics of the device. The light intensity was calibrated by using a reference cell with the KG5 window (Newport, USA). The *J–V* characteristics of the device were measured by using a Keithley 2400 SourceMeter (Tektronix, USA). There was no preconditioning to measure the device. The roughness and conductivity were measured by using atomic force microscopy in the contact mode (XE-100, Park systems, South Korea). Electrical properties of the perovskite microcells in Fig. 3h–k, such as PCEs of the perovskite microcells, were obtained by considering the AR of the microcells, i.e., by dividing the raw measurement data with the ratio of the microcell area to the entire device area. The microcell area is estimated by using SEM, i.e., by multiplying the area of one perovskite microcell measured by SEM (Supplementary Fig. 11b) with the number of total perovskite microcells. External quantum-efficiency (EQE) values were measured using a IQE-200B quantum-efficiency measurement system (Newport, USA). Electrical properties of the semitransparent PVs and colored solar windows in Figs. 4, 5 were measured as it is (i.e., raw measurement data).

### Optical characterization

The transmittance spectra of all fabricated samples were measured with a UV–Vis–NIR spectrometer (Cary 500, Varian, USA) using a tungsten–halogen lamp light source at a normal incidence-angle mode. The AVTs and CRIs of the semitransparent PVs and colored solar windows were calculated by considering photopic response curve of human eyes and solar photon flux^19,21^.

### Optical calculation

A rigorous coupled-wave analysis (RCWA) method was performed to calculate the light transmittance and absorptance of the semitransparent PVs and colored solar windows by using a commercial software (DiffractMOD, RSoft Design Group, USA). In the RCWA, the simulation condition was set with the second diffraction order and the grid size of 0.2 nm to calculate the diffraction efficiency, which was enough to numerically stabilize the calculation results. In addition, to obtain accurate outputs, material dispersions and extinction coefficients were considered. The commercial software, MATLAB (MathWorks, USA), was used to calculate the chromatic information from the transmittance data.

### Reporting summary

Further information on research design is available in the Nature Research Reporting Summary linked to this article.

## Supplementary information

Supplementary Information

Description of Additional Supplementary Files

Supplementary Movie 1

Solar Cells Reporting Summary

## Data Availability

All data are available in the main text or Supplementary Information.

## References

[1] Kim H-S (2012). Lead iodide perovskite sensitized all-solid-state submicron thin film mesoscopic solar cell with efficiency exceeding 9%. Sci. Rep..

[2] Yang WS (2015). High-performance photovoltaic perovskite layers fabricated through intramolecular exchange. Science.

[3] Tong J (2019). Carrier lifetimes of >1 μs in Sn-Pb perovskites enable efficient all-perovskite tandem solar cells. Science.

[4] Wehrenfenning C (2014). High charge carrier mobilities and lifetimes in organolead trihalide perovskites. Adv. Mater..

[5] Fang Y (2015). Highly narrowband perovskite single-crystal photodetectors enabled by surface-charge recombination. Nat. Photonics.

[6] Lin Q, Armin A, Burn PL, Meredith P (2015). Filterless narrowband visible photodetectors. Nat. Photonics.

[7] Du JS (2020). Halide perovskite nanocrystal arrays: Multiplexed synthesis and size-dependent emission. Sci. Adv..

[8] He X (2017). Patterning multicolored microdisk laser arrays of cesium lead halide perovskite. Adv. Mater..

[9] Xing D (2021). Self-healing lithographic patterning of perovskite nanocrystals for large-area single-mode laser array. Adv. Funct. Mater..

[10] Wang K (2020). Wettability-guided screen printing of perovskite microlaser arrays for current-driven displays. Adv. Maters..

[11] Wang YA (2019). Spin-on-patterning of Sn–Pb perovskite photodiodes on IGZO transistor arrays for fast active-matrix near-infrared imaging. Adv. Maters. Technol..

[12] Lee W (2017). High-resolution spin-on-patterning of perovskite thin films for a multiplexed image sensor array. Adv. Mater..

[13] National Renewable Energy Laboratory. “*Best research-cell efficiency chart*” https://www.nrel.gov/pv/cell-efficiency.html (2021).

[14] Eperon GE, Burlakov VM, Goriely A, Snaith HJ (2014). Neutral color semitransparent microstructured perovskite solar cells. ACS Nano.

[15] Eperon GE (2015). Efficient, Semitransparent neutral-colored solar cells based on microstructured formamidinium lead trihalide perovskite. J. Phys. Chem. Lett..

[16] Wu J (2017). Pinhole-free hybrid perovskite film with arbitrarily-shaped micro-patterns for functional optoelectronic devices. Nano Lett..

[17] Lee K (2020). Neutral-colored transparent crystalline silicon photovoltaics. Joule.

[18] Kang SB (2019). Stretchable and colorless freestanding microwire arrays for transparent solar cells with flexibility. Light Sci. Appl..

[19] Traverse CJ, Pandey R, Barr MC, Lunt RR (2017). Emergence of highly transparent photovoltaics for distributed applications. Nat. Energy.

[20] Lee K (2020). The development of transparent photovoltaics. Cell Rep. Phys. Sci..

[21] Yang C, Liu D, Lunt RR (2019). How to accurately report transparent luminescent solar concentrators. Joule.

[22] Fisette, P. “*Windows: understanding energy efficient performance*” https://bct.eco.umass.edu/publications/articles/windows-understanding-energy-efficient-performance/ (2003).

[23] Mescher J (2014). Design rules for semi-transparent organic tandem solar cells for window integration. Org. Electron..

[24] Lunt RR (2012). Theoretical limits for visibly transparent photovoltaics. Appl. Phys. Lett..

[25] Wang G (2015). Wafer-scale growth of large arrays of perovskite microplate crystals for functional electronics and optoelectronics. Sci. Adv..

[26] Lin C-H (2019). Orthogonal lithography for halide perovskite optoelectronic nanodevices. ACS Nano.

[27] Harwell J (2019). Patterning multicolor hybrid perovskite films via top-down lithography. ACS Nano.

[28] Waleed A (2017). Lead-free perovskite nanowire array photodetectors with drastically improved stability in nanoengineering templates. Nano lett..

[29] Gu L (2016). 3D arrays of 1024-pixel image sensors based on lead halide perovskite nanowires. Adv. Mater..

[30] Wang H (2016). Nanoimprinted perovskite nanograting photodetector with improved efficiency. ACS Nano.

[31] Alias MS (2016). Enhanced etching, surface damage recovery, and submicron patterning of hybrid perovskites using a chemically gas-assisted focused-ion beam for subwavelength grating photonic applications. J. Phys. Chem. Lett..

[32] Zou C (2020). Photolithographic patterning of perovskite thin films for multicolor display applications. Nano Lett..

[33] Park B-W (2018). Understanding how excess lead iodide precursor improves halide perovskite solar cell performance. Nat. Commun..

[34] Jang Y-W (2021). Intact 2D/3D halide junction perovskite solar cells via solid-phase in-plane growth. Nat. Energy.

[35] Watson BL, Rolston N, Printz AD, Dauskardt RH (2017). Scaffold-reinforced perovskite compound solar cells. Energy Environ. Sci..

[36] Rolston N (2018). Effect of cation composition on the mechanical stability of perovskite solar cells. Adv. Energy Mater..

[37] Song YM, Jang SJ, Yu JS, Lee YT (2010). Bioinspired parabola subwavelength structures for improved broadband antireflection. Small.

[38] Park S (2018). Self-powered ultra-flexible electronics via nano-grating-patterned organic photovoltaics. Nature.

[39] Leem JW (2014). Efficiency enhancement of organic solar cells using hydrophobic antireflective inverted moth-eye nanopatterned PDMS films. Adv. Energy Maters..

[40] Kim M (2019). Moth-eye structured polydimethylsiloxane films for high-efficiency perovskite solar cells. Nano-Micro Lett..

[41] Kim DH, Yoo YJ, Ko JH, Kim YJ, Song YM (2019). Standard red green blue (sRGB) color representation with a tailored dual-resonance mode in metal/dielectric stacks. Opt. Mater. Express.

[42] Munoz P (2019). Double metal layer lift-off process for the robust fabrication of plasmonic nano-antenna arrays on dielectric substrates using e-beam lithography. Optical Mater. Express.

[43] Yi C (2016). Entropic stabilization of mixed A-cation ABX3 metal halide perovskites for high performance perovskite solar cells. Energy Environ. Sci..

[44] Coburn JW, Winters HF (1979). Ion‐ and electron‐assisted gas‐surface chemistry—An important effect in plasma etching. J. Appl. Phys..

[45] Kim H, Lim K-G, Lee T-W (2016). Planar heterojunction organometal halide perovskite solar cells: roles of interfacial layers. Energy Environ. Sci..

[46] Bett AJ (2019). Semi-transparent perovskite solar cells with ITO directly sputtered on Spiro-OMeTAD for tandem applications. ACS Appl. Mater. Interfaces.

[47] Yanoff, M. & Duker, J. S. *Ophthalmology: Expert Consult* 3rd ed. (Mosby Elsevier, 2009).

[48] Yoo YJ (2019). Mechanically robust antireflective moth-eye structures with a tailored coating of dielectric materials. Opt. Mater. Express.

[49] Gomard G (2010). Two-dimensional photonic crystal for absorption enhancement in hydrogenated amorphous silicon thin film solar cells. J. Appl. Phys..

[50] Yeom HR (2020). Aesthetic and colorful: Dichroic polymer solar cells using high-performance Fabry-Perot etalon electrodes with a unique Sb2O3 cavity. Nano Energy.

[51] Chen D (2019). Dynamic tunable color display based on metal–insulator–metal resonator with polymer brush insulator layer as signal transducer. ACS Appl. Mater. interfaces.

[52] Camgöz N, Yener C, Güvenç D (2002). Effects of hue, saturation, and brightness on preference. Color Res. Appl..

