# Supplementary material for: Perovskite microcells fabricated using swelling-induced crack propagation for colored solar windows
Source: Nat Commun. 2022 Apr 11;13:1946. doi: 10.1038/s41467-022-29602-z (PMC9001655; doi:10.1038/s41467-022-29602-z)
Supplement: Supplementary file 1 — Supplementary Information [file 41467_2022_29602_MOESM1_ESM.pdf]

Supplementary Information for

Perovskite microcells fabricated using swelling-induced crack propagation for colored solar windows

Woongchan Lee[†], Young Jin Yoo[†], Jinhong Park[†], Joo Hwan Ko, Yeong Jae Kim, Huiwon Yun, Dong Hoe Kim[*], Young Min Song[*], and Dae-Hyeong Kim[*]

[†]These authors contributed equally to this work.
[*]Correspondences to: dkim98@snu.ac.kr, ymsong@gist.ac.kr, and donghoekim@korea.ac.kr

This PDF file includes:

Supplementary Figures 1 to 24
Supplementary Table 1 to 4
Supplementary References 1 to 61

Other Supplementary Information for this manuscript include the following:

Supplementary Movie 1

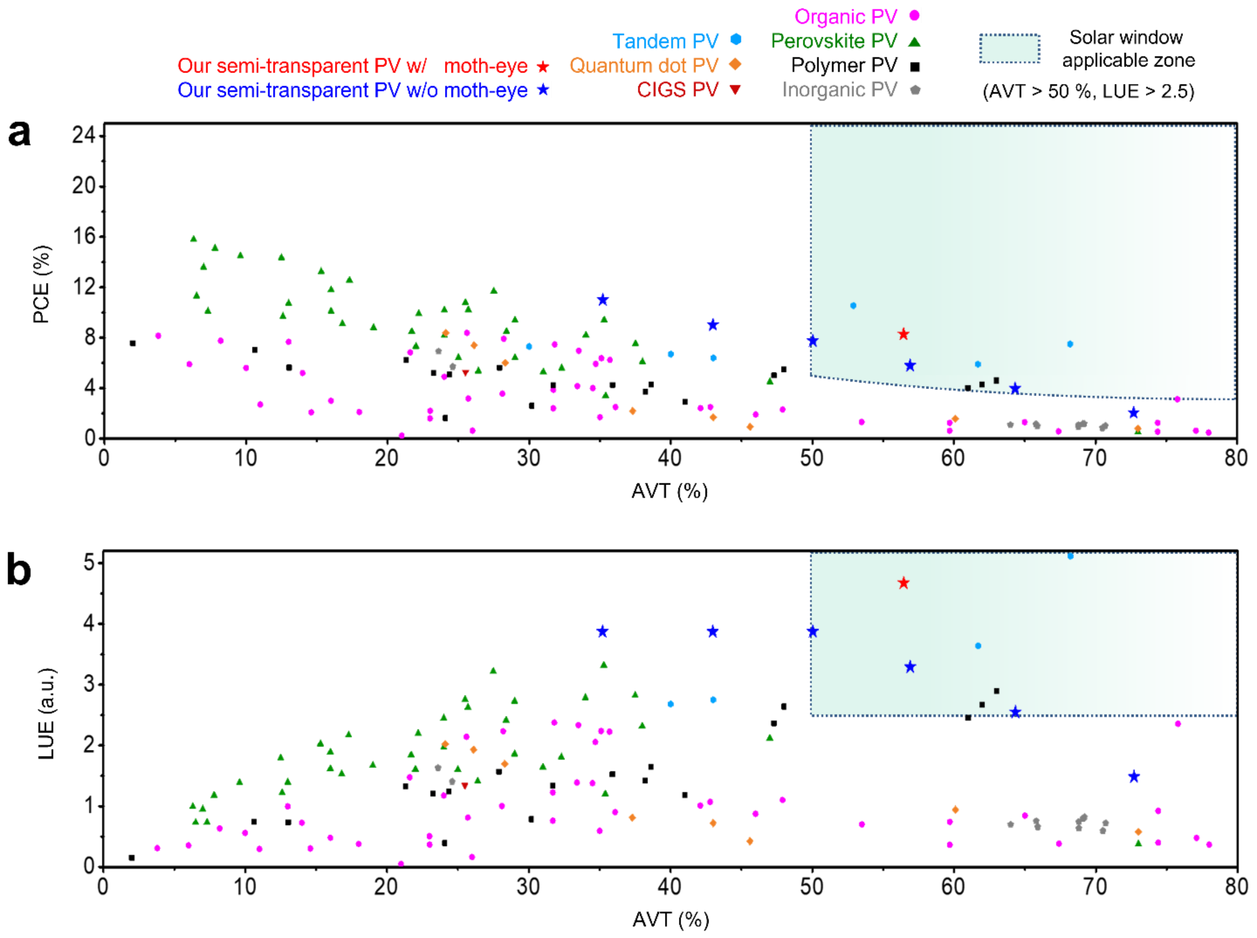

Supplementary Fig. 1 | Comparison of power conversion efficiencies (PCEs) and average visible transmittances (AVTs) of semi-transparent photovoltaic devices (PVs). a, PCEs and AVTs of semi-transparent PVs fabricated by the proposed method with the moth-eye nanostructure (red star) and without the moth-eye nanostructure (blue star) are plotted. Reference data[1-49] from previous reports for other types of semi-transparent PVs, such as tandem PVs (blue pentagon), quantum dot PVs (orange rhombus), copper indium gallium selenide (CIGS) PVs (red inverted triangle), organic PVs (pink circle), perovskite PVs (green triangle), polymer PVs (black rectangle), and inorganic PVs (gray pentagon), are co-plotted for comparison. **b,** Light utilization efficiencies (LUEs = AVT × PCEs) calculated from data in (a) are plotted to evaluate AVT and PCE simultaneously.

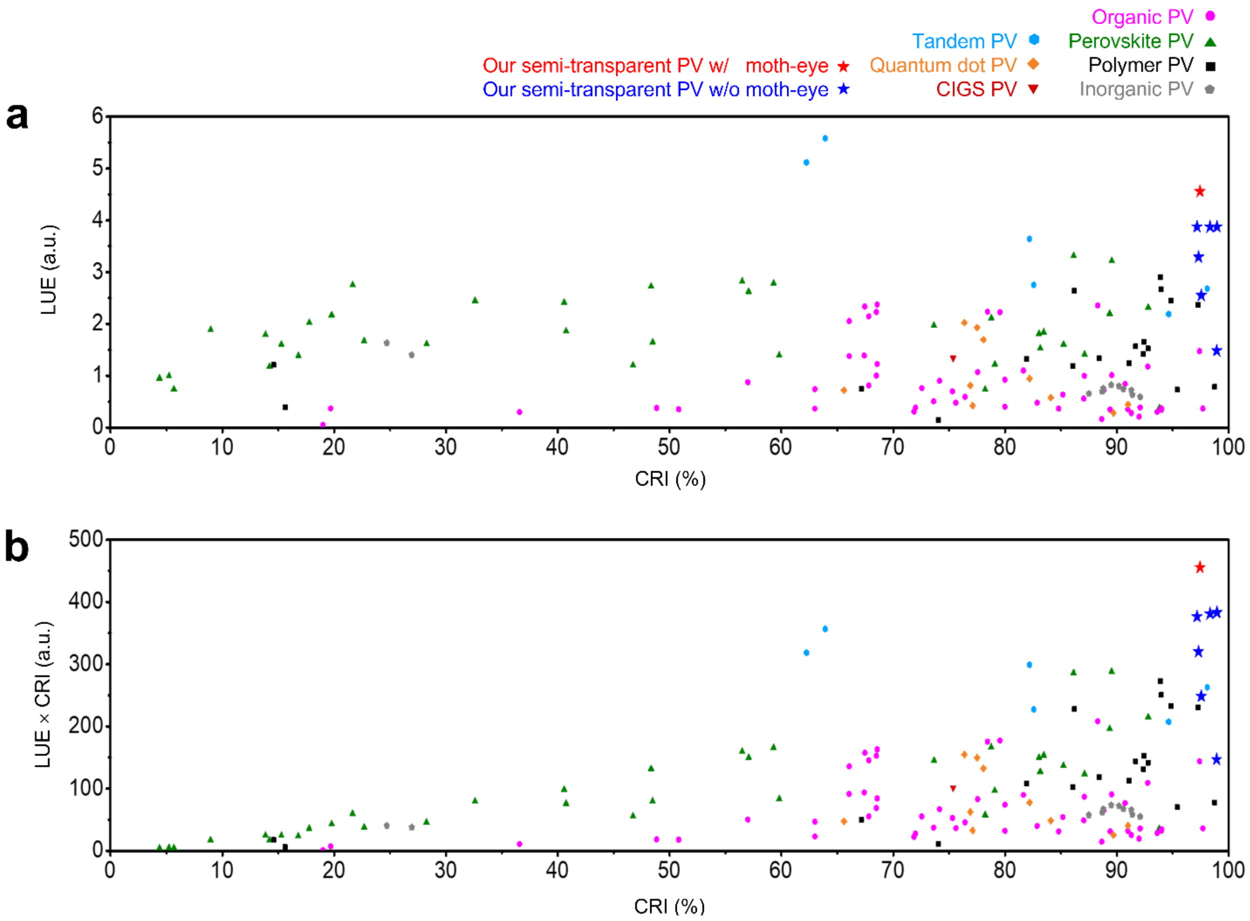

Supplementary Fig. 2 | Comparison of LUEs and color rendering indices (CRIs) of semi-transparent PVs. a, CRIs and LUEs of semi-transparent PVs fabricated by the proposed method with the moth-eye nanostructure (red star) and without the moth-eye nanostructure (blue star) are plotted. Reference data[1-49] from previous reports for other types of semi-transparent PVs, such as tandem PVs (blue pentagon), quantum dot PVs (orange rhombus), copper indium gallium selenide (CIGS) PVs (red inverted triangle), organic PVs (pink circle), perovskite PVs (green triangle), polymer PVs (black rectangle), and inorganic PVs (gray pentagon), are co-plotted for comparison. **b,** LUE × CRIs calculated from data in (a) are plotted to evaluate LUE and CRI, simultaneously. The CRIs not included in the original reference data were calculated.[50]

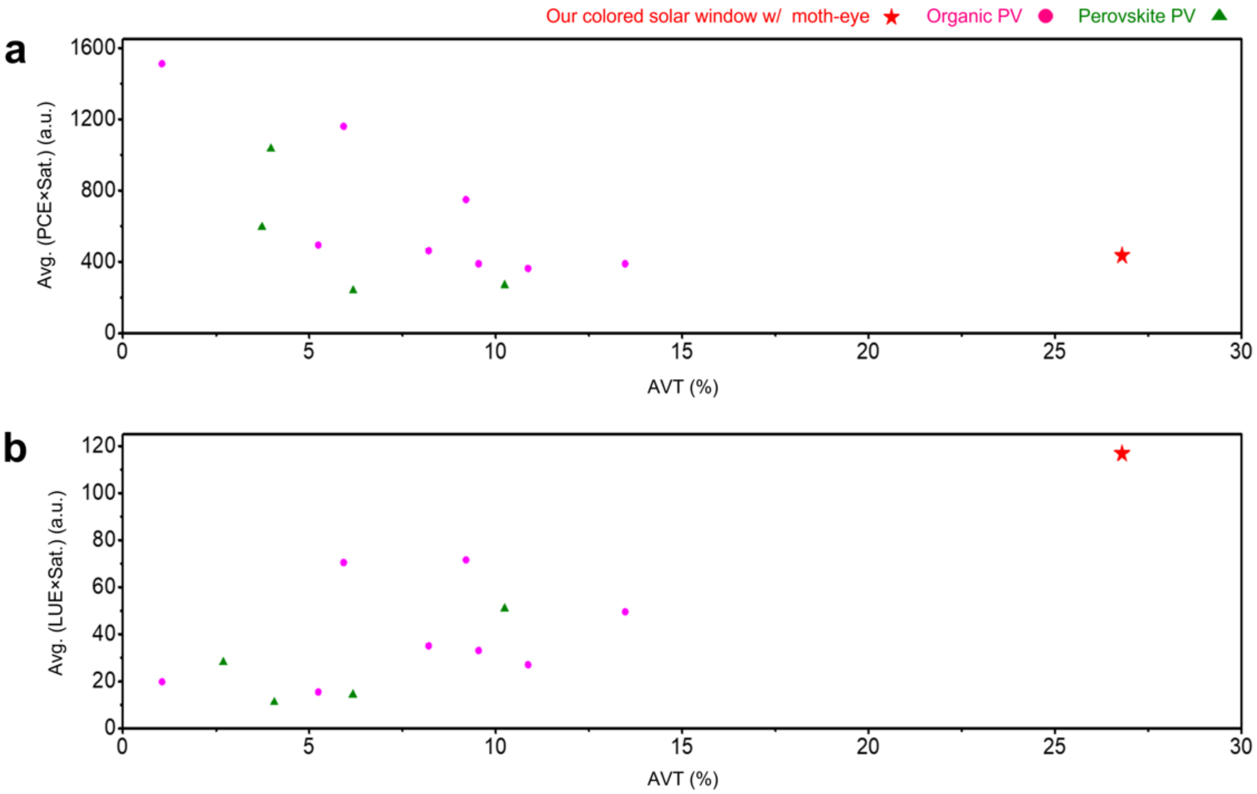

Supplementary Fig. 3 | Comparison of chromatic saturations, PCEs, and AVTs of colored PVs. a, PCE × chromatic saturation (Saturation) versus AVT of the colored PV fabricated by the proposed method with the moth-eye nanostructure (red star) is plotted. Reference data[51-58] from previous reports for other types of transmissive colored PVs, such as organic PVs (pink circle) and perovskite PVs (green triangle), are co-plotted for comparison. PCE × Saturation values were calculated as an average value for different colors (*i.e.*, red, green, and blue). **b**, LUE × Saturation values calculated from data in (a) are plotted according to AVTs to evaluate AVT, PCE, and Saturation, simultaneously.

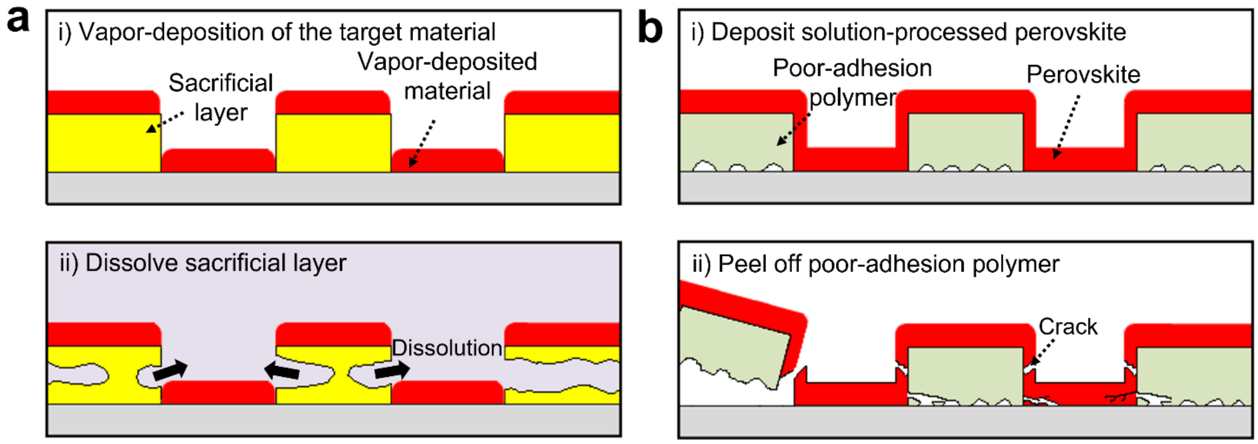

Supplementary Fig. 4 | Schematic illustrations that explain various lift-off processes. a, Lift-off process for the vapor-deposited material. i) Vapor-deposition of the target material over the patterned sacrificial layer. The sidewall of the sacrificial layer is not covered by the vauum-deposited material. ii) Dissolution of the sacrificial layer, such as photoresists, in the solvent is facile. **b,** Lift-off process for the solution-processed perovskite thin film. i) Deposit the solution-processed perovskite film over the poor-adhesion polymer. The sidewall of the sacrificial layer is covered by the solution-based material. Therefore, instead of dissolving the sacrificial layer in the solvent, a sacrificial polymer whose adhesion to the substrate is poor is typically used. ii) Mechanical peeling-off procedure of the poor-adhesion polymer from the bottom substrate to pattern the perovskite film. During the peeling-off procedure, cracks, fractures, and partial delamination occur in the patterned perovskite film.

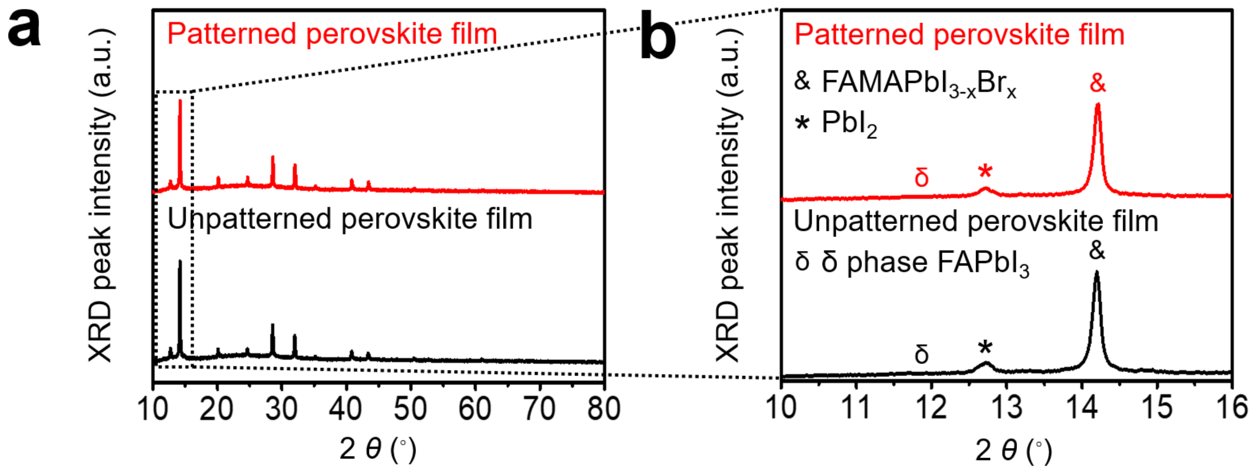

Supplementary Fig. 5 | X-ray diffraction (XRD) analysis of the patterned perovskite film and the unpatterned perovskite film. a, XRD pattern of the patterned perovskite film (top) and the unpatterned perovskite film (bottom). **b,** Magnified view of the XRD pattern in (a) to show the detailed peak information. The ratio of $FAMAPbI_{3-x}Br_x$ and PbI_2 in the XRD pattern of the patterned perovskite film is same as that of the unpatterned perovskite film. In addition, δ phase $FAPbI_3$ is not shown in both films.

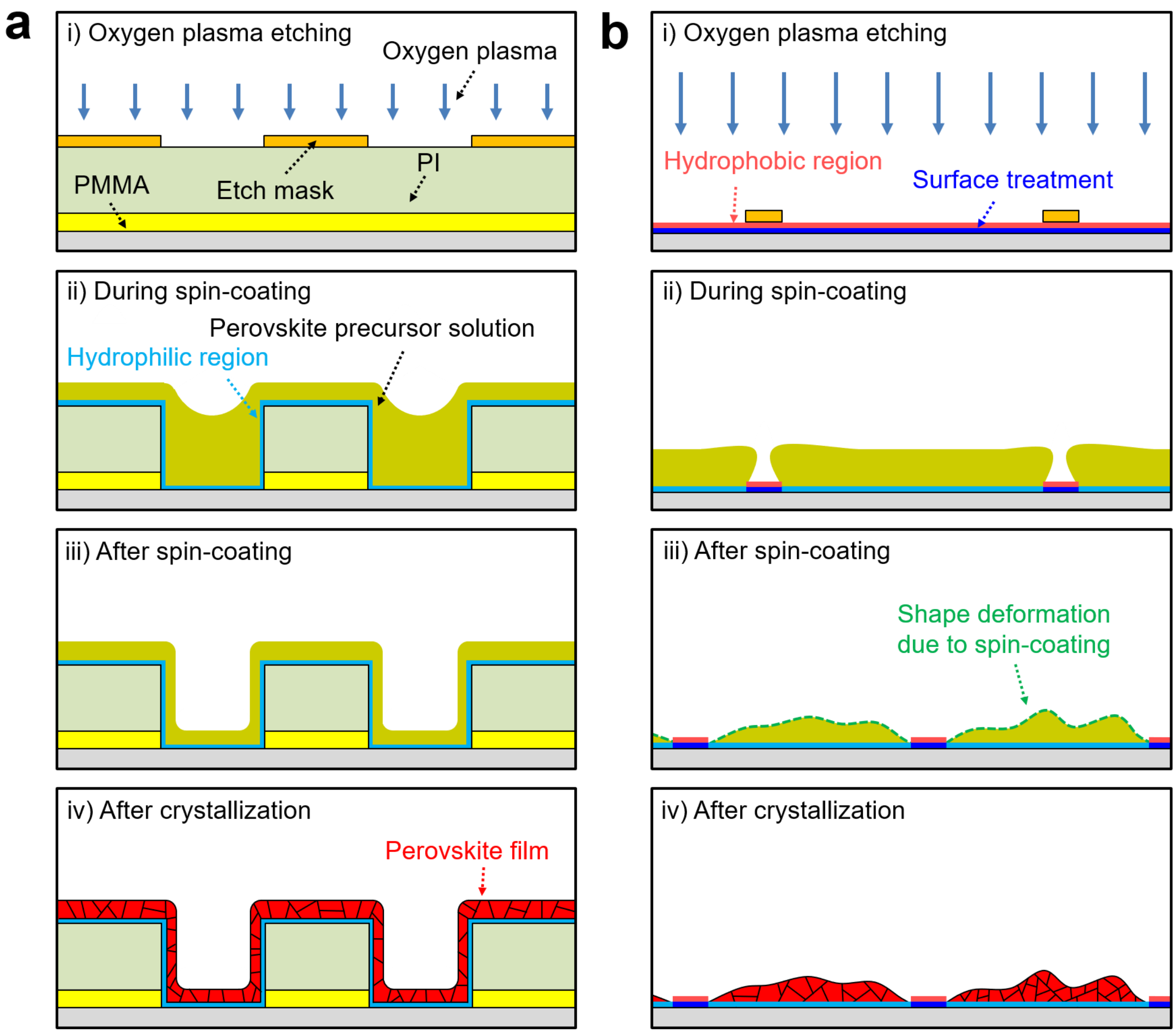

Supplementary Fig. 6 | Step-by-step cross-sectional illustrations of two kinds of perovskite patterning methods. a, Step-by-step cross-sectional illustrations of the lift-off method. After the oxygen plasma etching, the surface property becomes hydrophilic. Therefore, the thickness of the coated perovskite film is uniform, and the surface of the film is flat. **b,** Step-by-step cross-sectional illustrations of the dewetting method.[59] The film thickness is not uniform, and the surface of the patterned film is not flat. The edge of the patterned film can be thinner than the center, which decreases the volume of the perovskite precursor solution and leads to the defective and deficient crystal growth.

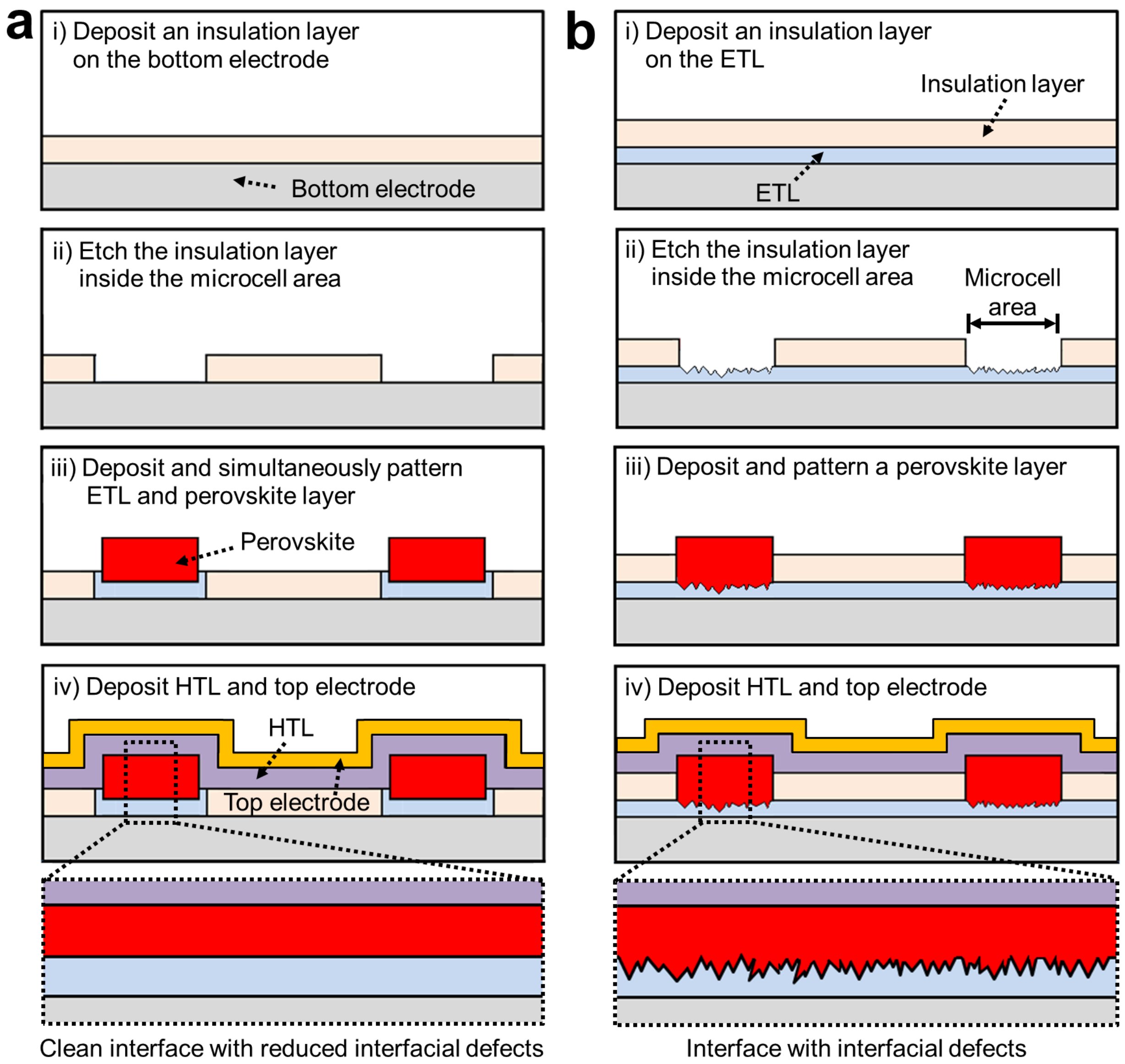

Supplementary Fig. 7 | Comparison of the simultaneous lift-off structure and the conventional structure in terms of the interfacial defect between the perovskite layer and the electron transport layer (ETL). a, Schematic illustrations of the fabrication process and the interface quality of the simultaneous lift-off structure. The insulation layer is etched before the deposition of the ETL and the perovskite layer. This simultaneous patterning of the ETL and the perovskite layer enables to suppress interfacial defects between the ETL and the perovskite layer. **b,** Schematic illustrations of the fabrication process and the interface quality of the conventional structure. During the etching process of the insulation layer (beige), the surface of the ETL (blue) is damaged, regardless of using dry etching or wet etching methods, and thus the interface between the ETL and the perovskite layer (red) has more interfacial defects.[60,61] Note that the simultaneous lift-off structure needs to use the lift-off method, while the conventional structure can be fabricated by either the lift-off method or the dewetting method.

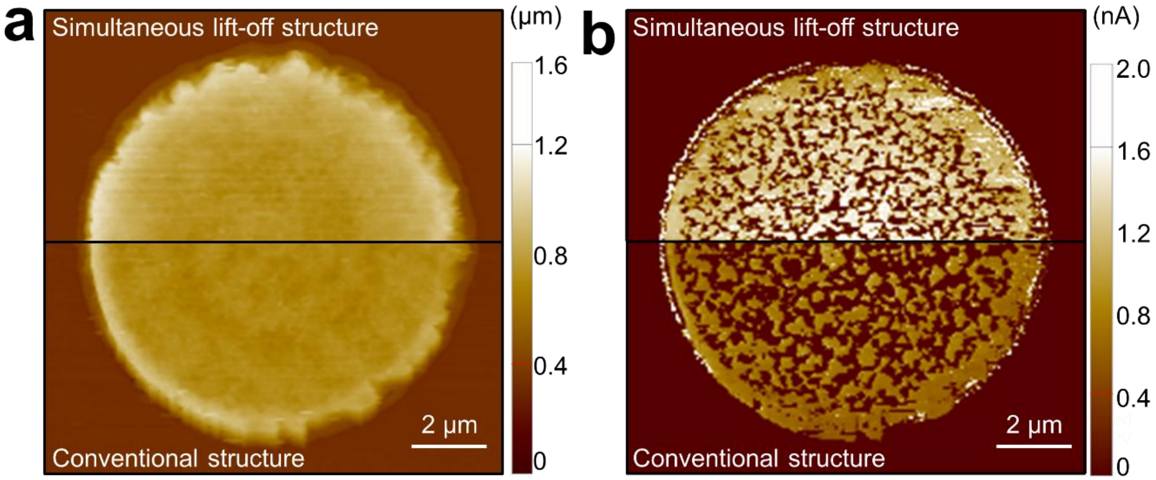

Supplementary Fig. 8 | Comparison of the simultaneous lift-off structure and the conventional structure in terms of surface morphology. a, Surface morphology measured by AFM for the ETL/perovskite layer in the simultaneous lift-off structure (top) and the conventional structure (bottom). **b,** Conductive AFM measurement of the ETL/perovskite layer in the simultaneous lift-off structure (top) and the conventional structure (bottom).

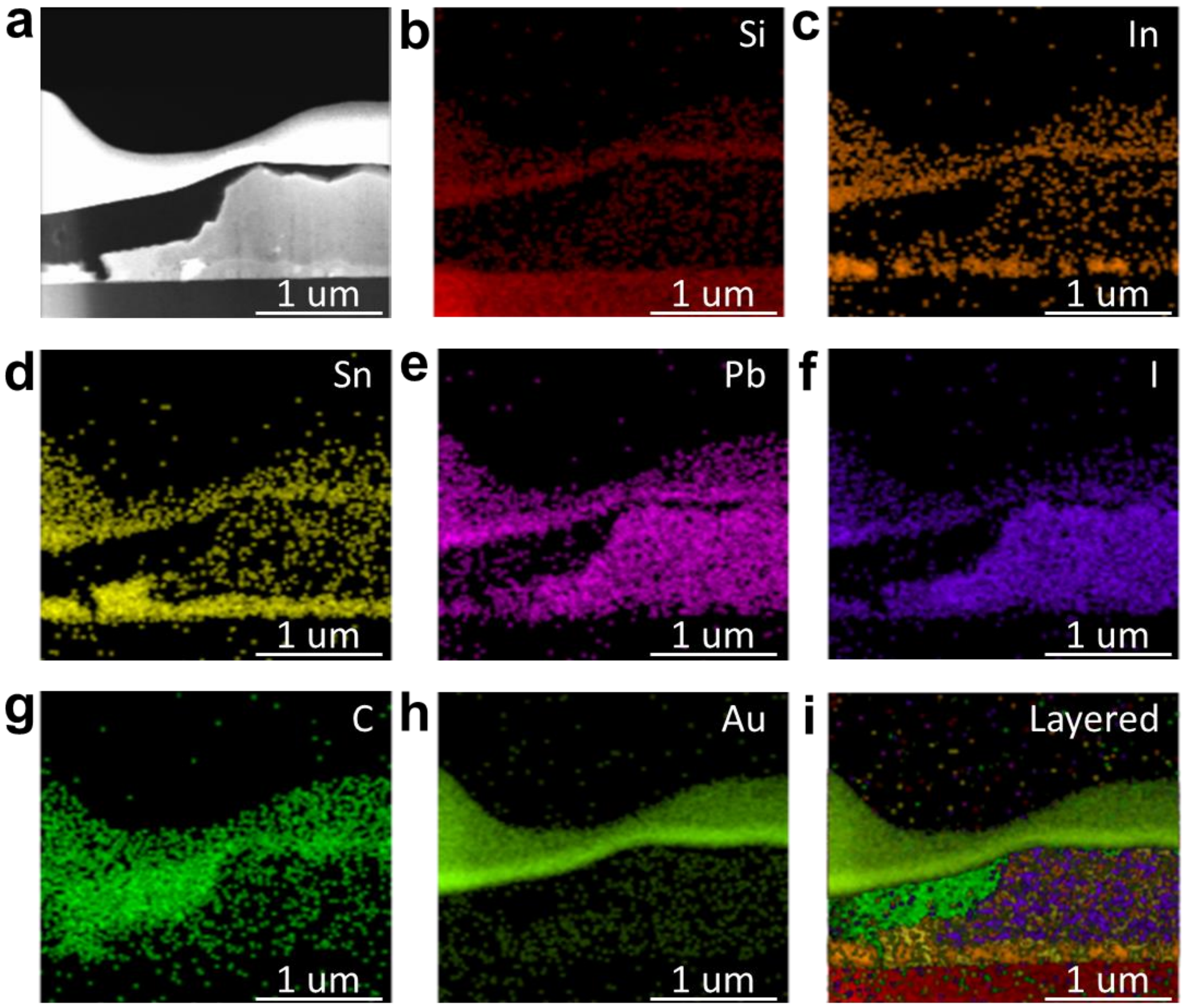

Supplementary Fig. 9 | Cross-sectional transmission electron microscopy (TEM) image and energy dispersive spectroscopy (EDS) analysis of the perovskite microcell. a, Normal TEM image of the edge morphology in the perovskite microcell. Elemental distribution mapping of **b**, silicon, **c**, indium, **d**, tin, **e**, lead, **f**, iodine, **g**, carbon, **h**, gold through the EDS analysis and **i**, layered mapping image.

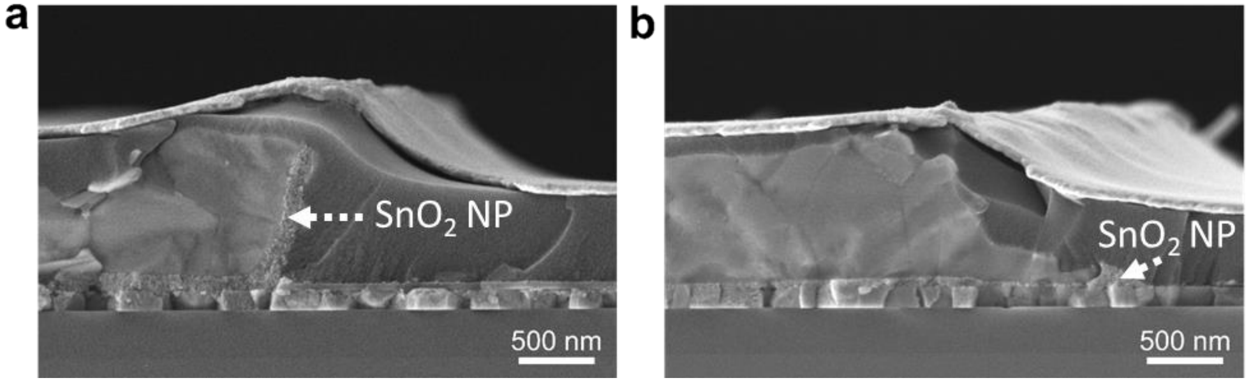

Supplementary Fig. 10 | Effects of fabrication condition on the device structures. Scanning electron microscopy (SEM) images of device structures with **a**, dry etching using O_2 plasma and **b**, wet etching by using a buffered oxide etchant.

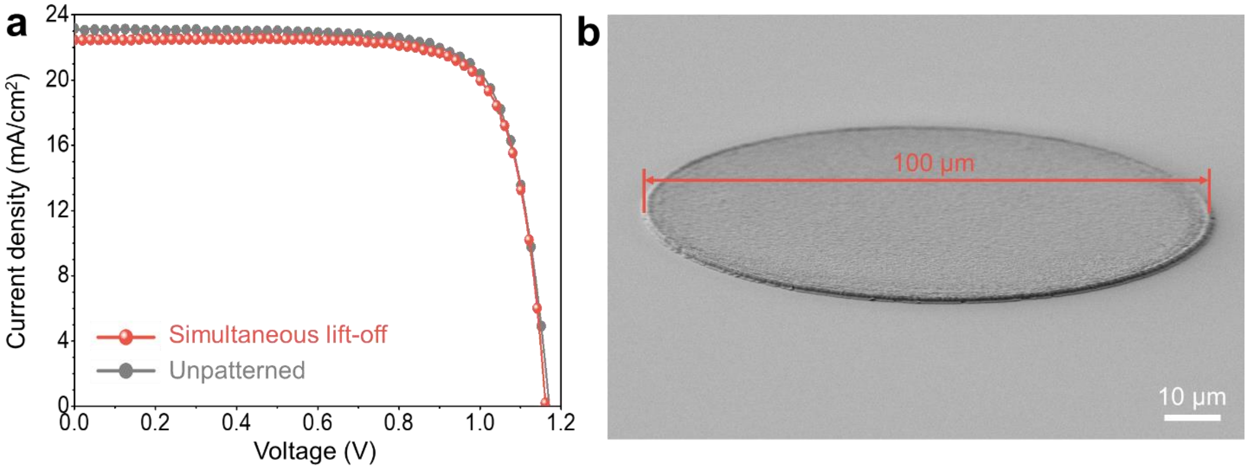

Supplementary Fig. 11 | Comparison of the current density and voltage (*J*–*V*) characteristics between the perovskite microcell and the unpatterned perovskite PV and measurement of the perovskite microcell's diameter. a, Comparison of *J-V* characteristics of the perovskite microcell (simultaneous lift-off structure) and that of the unpatterned perovskite PV. **b,** SEM image of a perovskite microcell for the measurement of its diameter and the calculation of the microcell area.

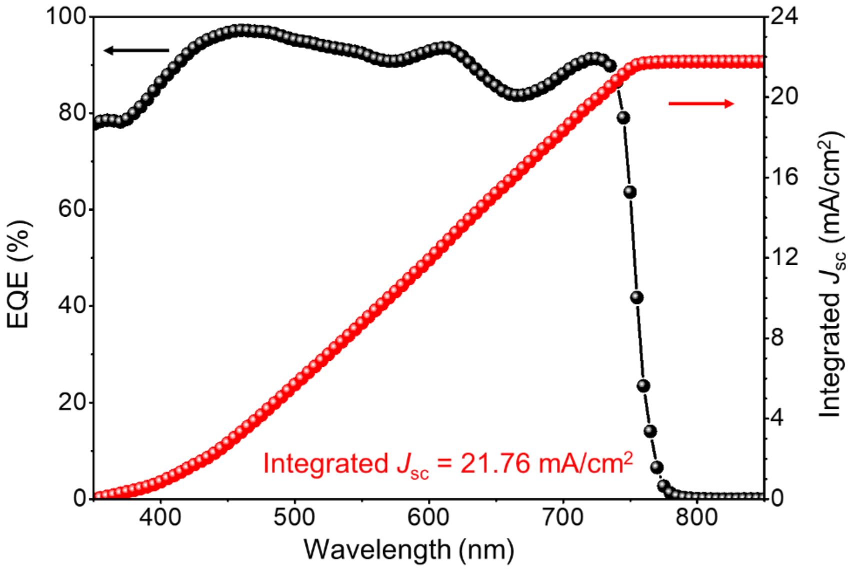

Supplementary Fig. 12 | Optoelectrical properties of the perovskite microcell. External quantum efficiency and integrated J_{sc} of the perovskite microcell with the simultaneous lift-off structure.

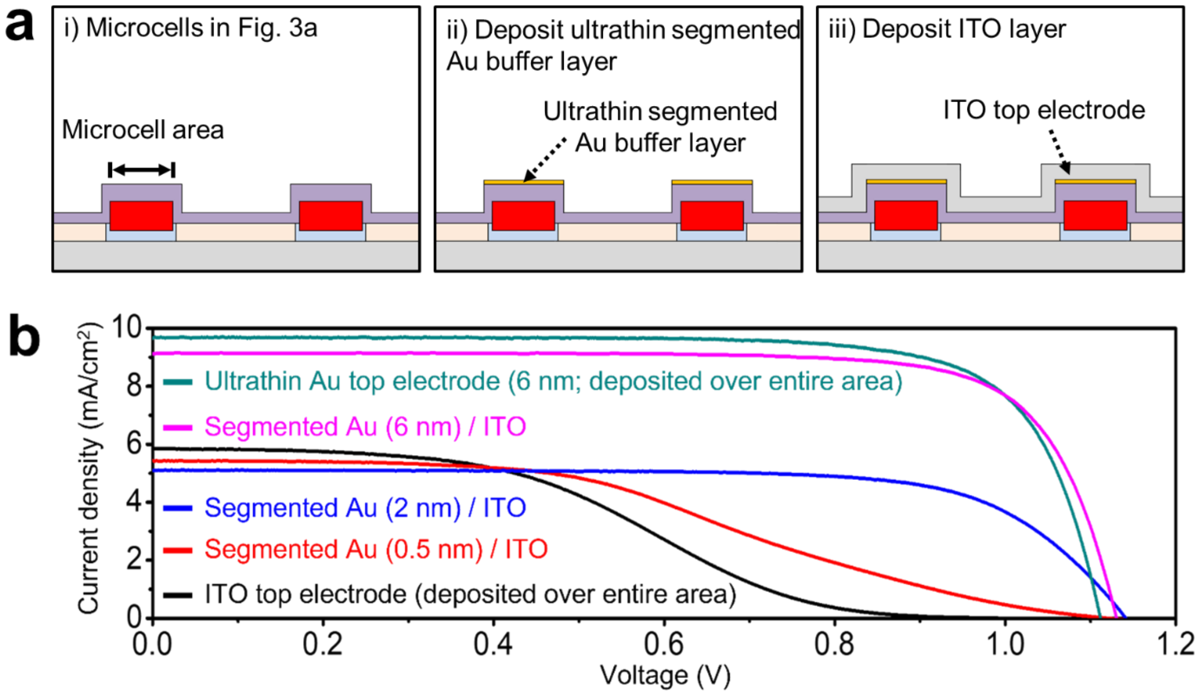

Supplementary Fig. 13 | Schematic illustrations for the fabrication process of the semi-transparent PV and electrical properties of the semi-transparent PVs with various types of buffer layers and top electrodes. a, Cross-sectional schematic illustrations that show the fabrication process of the semi-transparent PV. i) patterning of perovskite microcells before the deposition of the top electrode, ii) selective deposition of an ultrathin segmented Au buffer layer (6 nm; yellow) inside the microcell area, and iii) deposition of an ITO top electrode (150 nm; grey) over the entire device area. **b,** *J*–*V* characteristics of the semi-transparent PVs with various thicknesses of the segmented Au buffer layer (0 nm (black), 0.5 nm (red), 2 nm (blue), and 6 nm (magenta)) and the ITO top electrode (150 nm). *J*–*V* characteristics of the perovskite microcells with the ultrathin Au top electrode (6 nm; cyan; deposited over the entire device area) are co-plotted for comparison. The results were measured under the reverse scan.

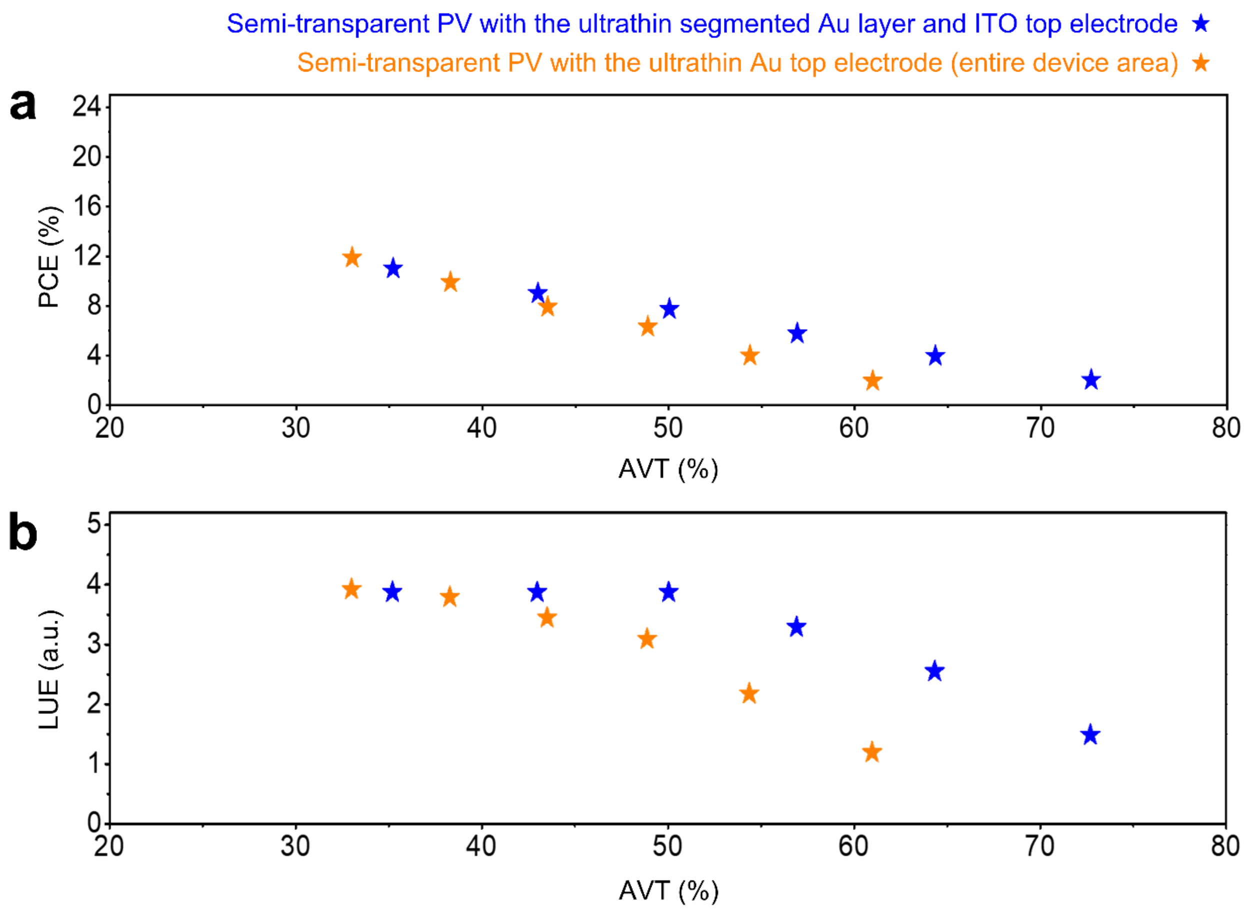

Supplementary Fig. 14 | Comparison of power conversion efficiencies and average visible transmittances of the semi-transparent PVs composed of perovskite microcells with the ultrathin segmented Au buffer layer and ITO top electrode and those composed of perovskite microcells with the ultrathin Au top electrode (entire device area). a, Plot of PCEs versus AVTs for the semi-transparent PVs composed of perovskite microcells with the ultrathin segmented Au buffer layer and ITO top electrode (blue star) and PCEs versus AVTs for the semi-transparent PVs composed of perovskite microcells with the ultrathin Au top electrode (orange star; deposited over the entire device area). **b,** LUEs of the semi-transparent PVs, which are calculated by using data presented in (a).

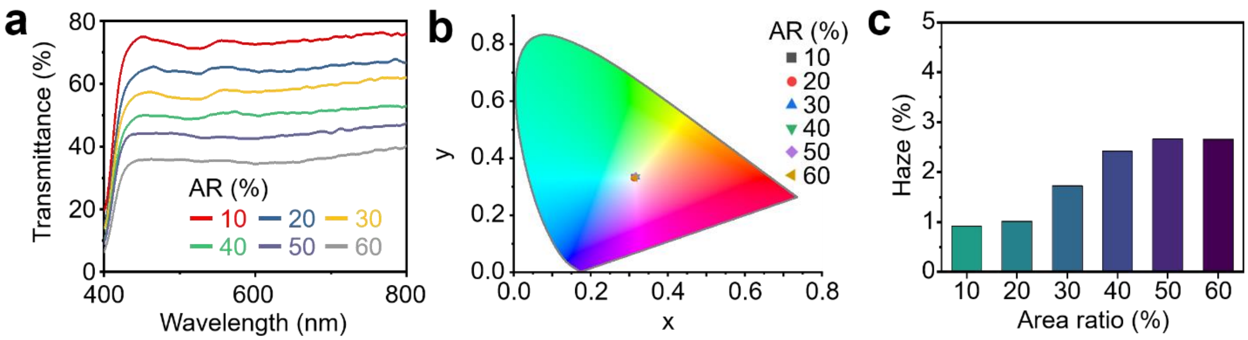

Supplementary Fig. 15 | Optical properties of semi-transparent PVs with various area ratios (ARs). a, Transmittance spectra and **b**, CIE coordinate of semi-transparent PVs with various ARs. **c**, Haze of the semi-transparent PVs with various ARs.

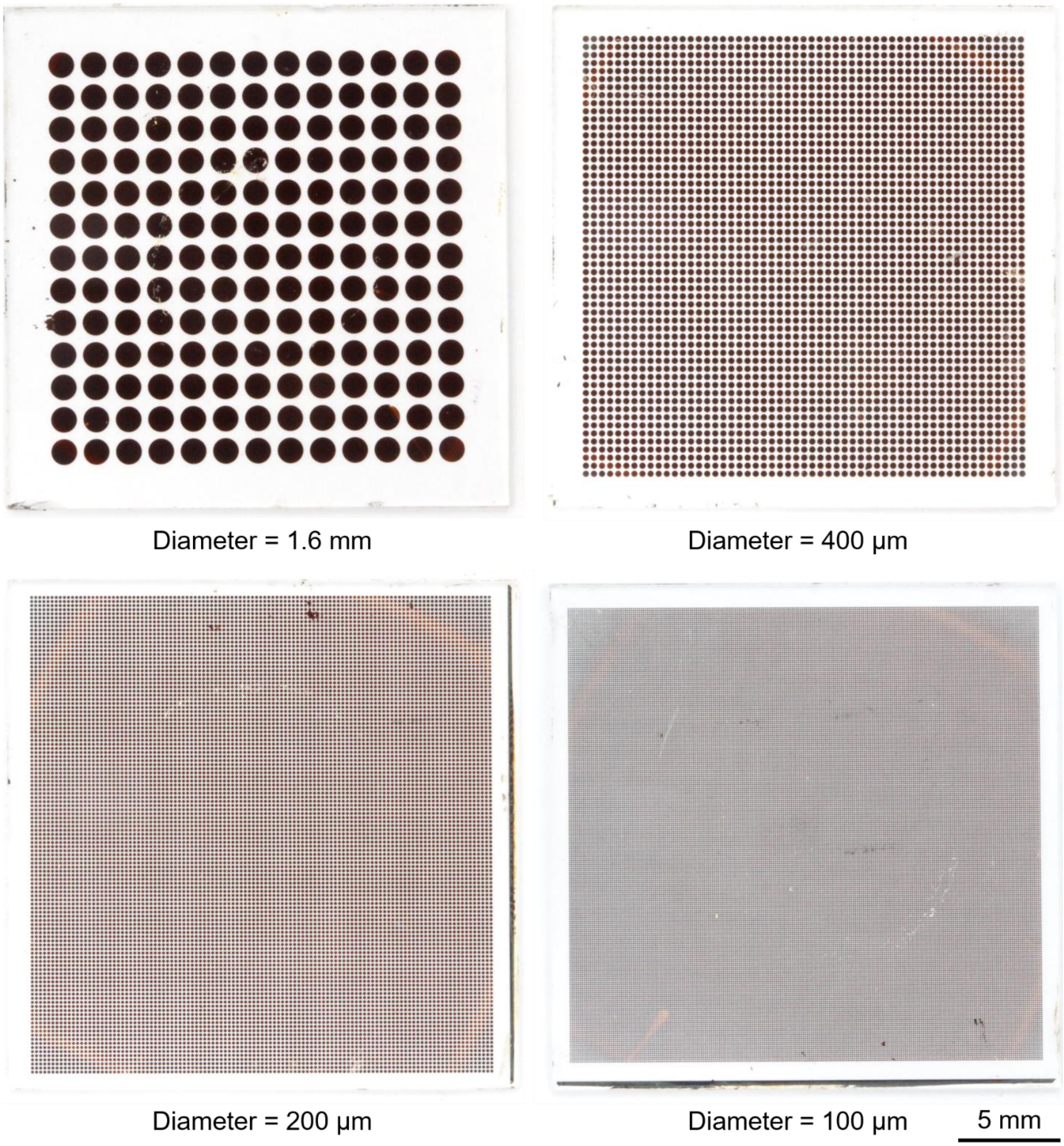

Supplementary Fig. 16 | Photographs of the perovskite PVs with various diameters of the perovskite microcells. The diameter of each perovskite microcell is 1.6 mm, 400 μm, 200 μm, and 100 μm. Images were magnified to show the pattern (refer to the scale bar).

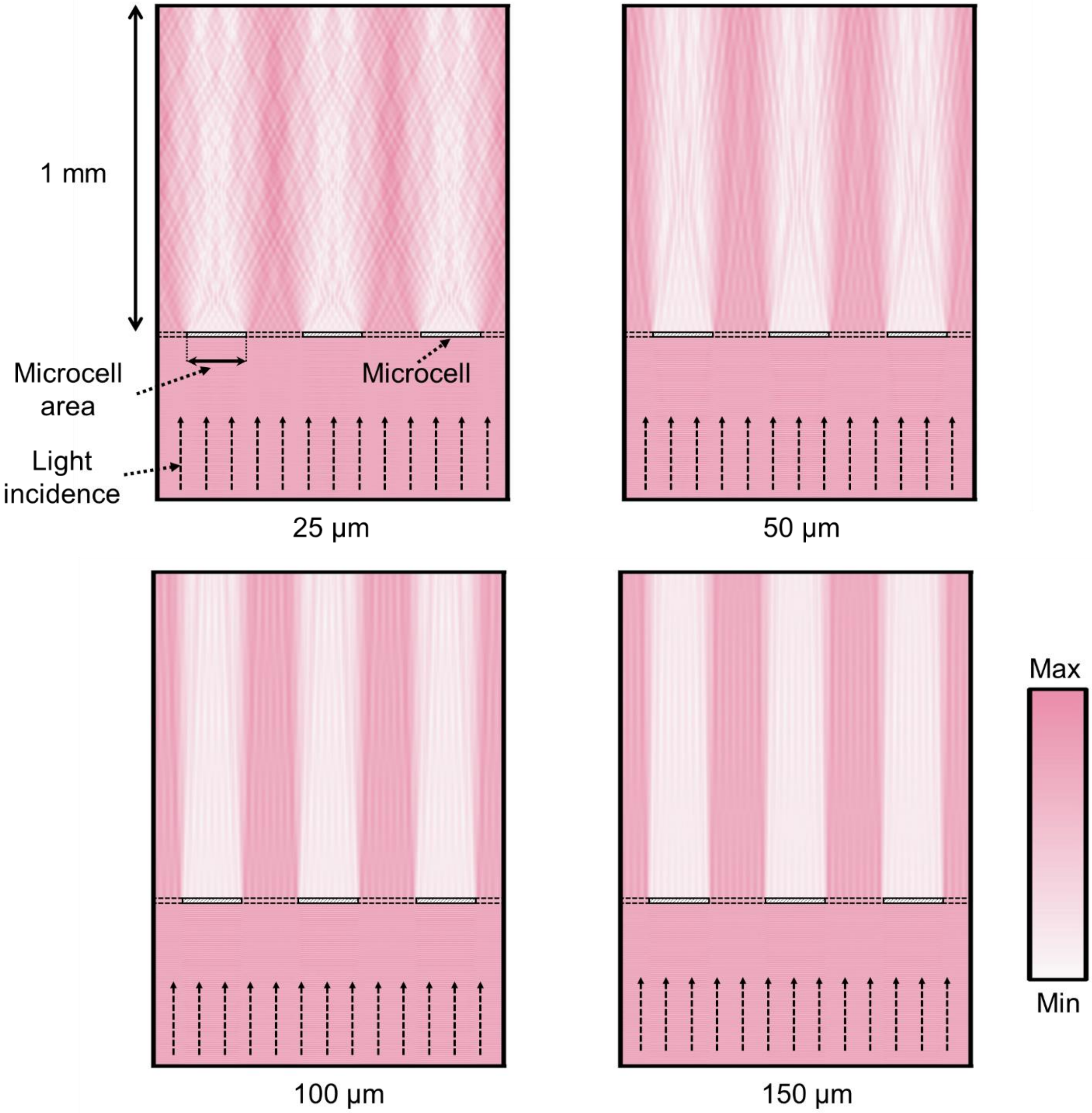

Supplementary Fig. 17 | Electric field distribution of light transmitted through semi-transparent PVs. The diameter of perovskite microcells is varied from 25 μm to 150 μm under a fixed ratio of the microcell area to the entire device area. The haze is severe if the diameter of perovskite microcells is decreased < 50 μm.

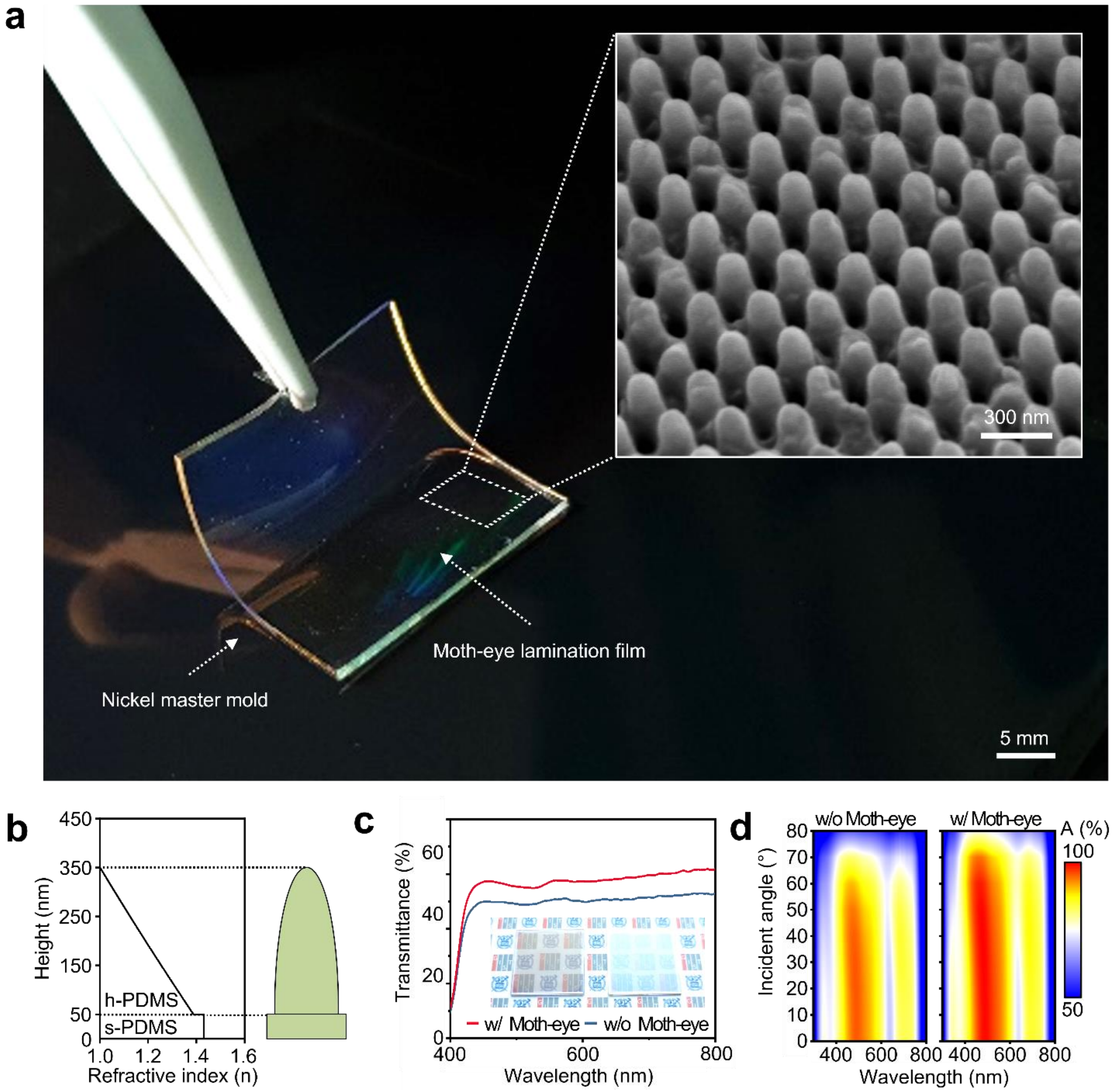

Supplementary Fig. 18 | Moth-eye-inspired nanostructure and its effects on improving optical properties. a, Optical camera image of a film with the moth-eye-inspired nanostructure. The film is made of s-PDMS and h-PDMS and fabricated by using the nanoimprint method with a nickel master mold. The inset shows a SEM image of the fabricated moth-eye nanostructure. **b,** Gradual refractive index profile (left) of the moth-eye nanostructure (right). **c,** Transmittance measurement of the semi-transparent PV with and without the moth-eye nanostructures. The inset shows photographs of semi-transparent PVs with and without the moth-eye nanostructures. **d,** Calculated light absorptance of the semi-transparent PV with and without the moth-eye nanostructures for various wavelengths and light-incident angles.

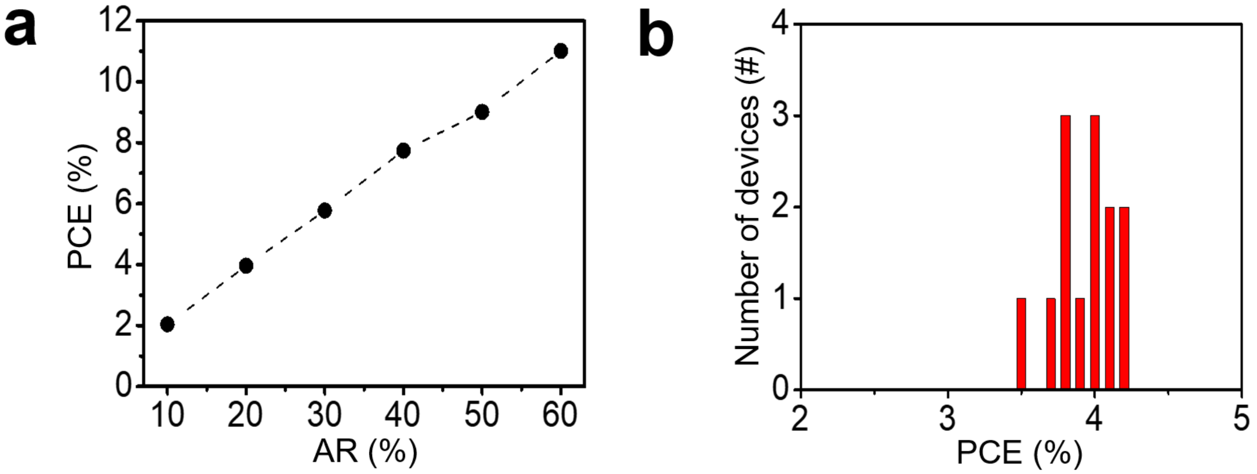

Supplementary Fig. 19 | PCEs of the semi-transparent PVs. a, PCEs (reverse scan) of the semi-transparent PVs for various ARs. **b,** Statistical data on the PCE (reverse scan) of semi-transparent PVs with an AR of 20 %.

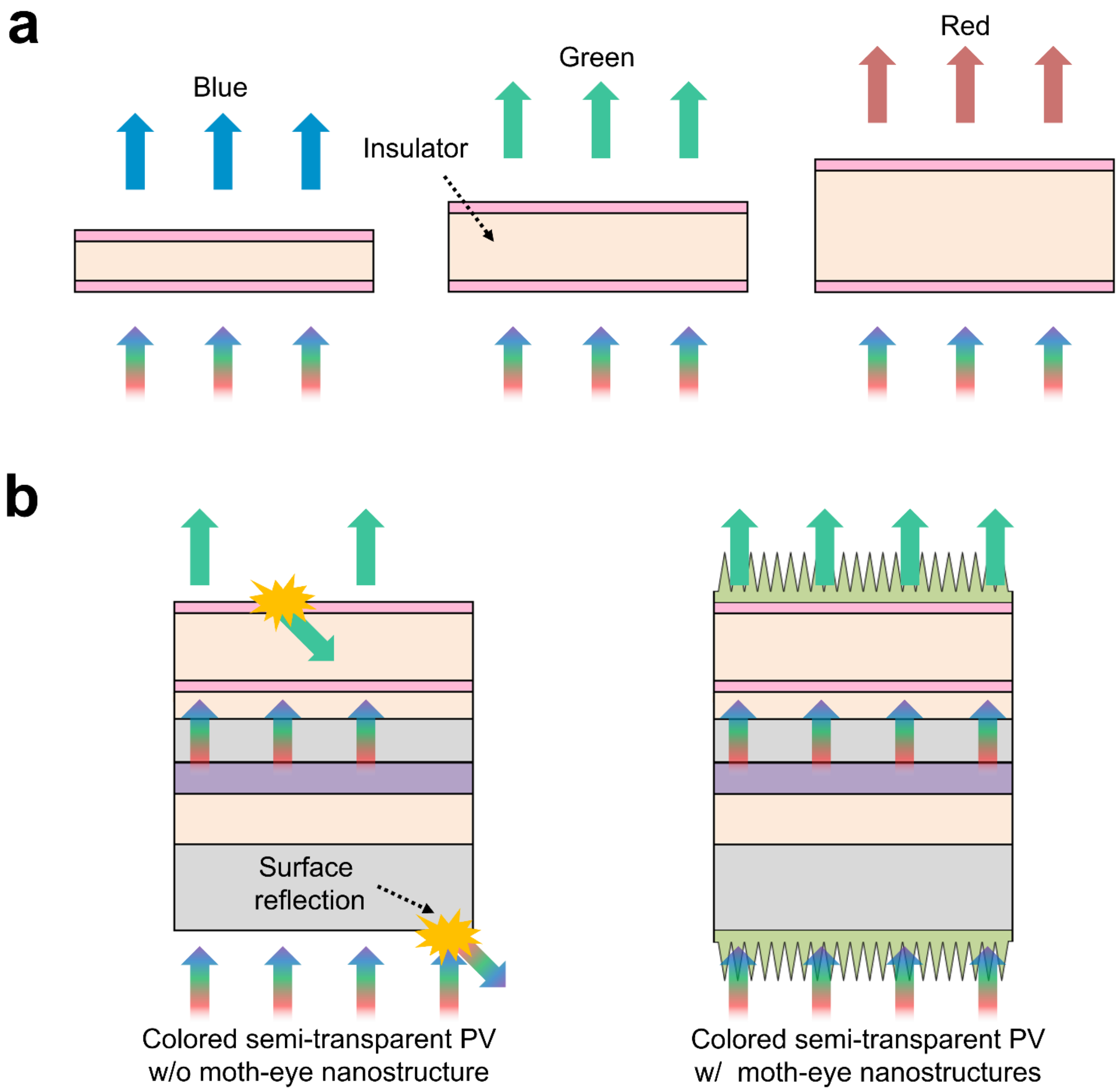

Supplementary Fig. 20 | Roles of MIM structure and moth-eye nanostructures in the colored solar window. a, Schematic illustrations that show the selective light transmission through the MIM structure depending on the thickness of the insulator inside the MIM structure. **b,** Schematic illustrations that explain the enhancement of the chromatic transmittance by the MIM structure with the moth-eye nanostructures at the outside of the microcell area.

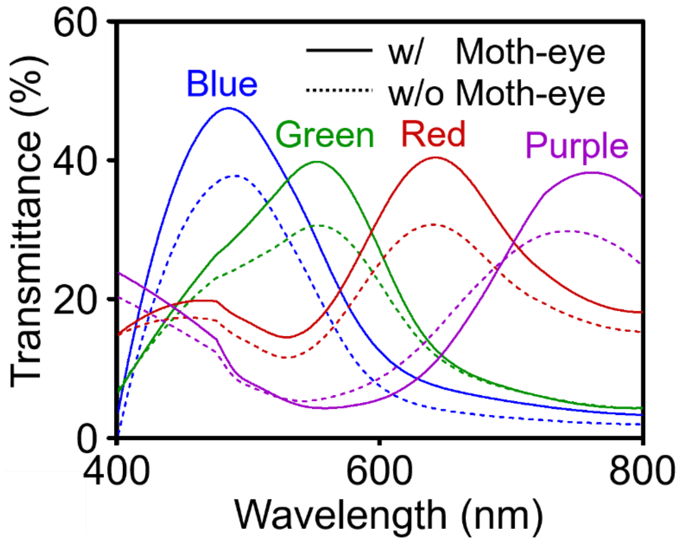

Supplementary Fig. 21 | Light transmittance of the colored solar window with and without the moth-eye nanostructures. Transmittance spectra of colored solar windows with and without the moth-eye nanostructures are plotted for the blue, green, red, and purple MIM structures. The colored solar windows were fabricated using the semi-transparent PVs with an AR of 40 %.

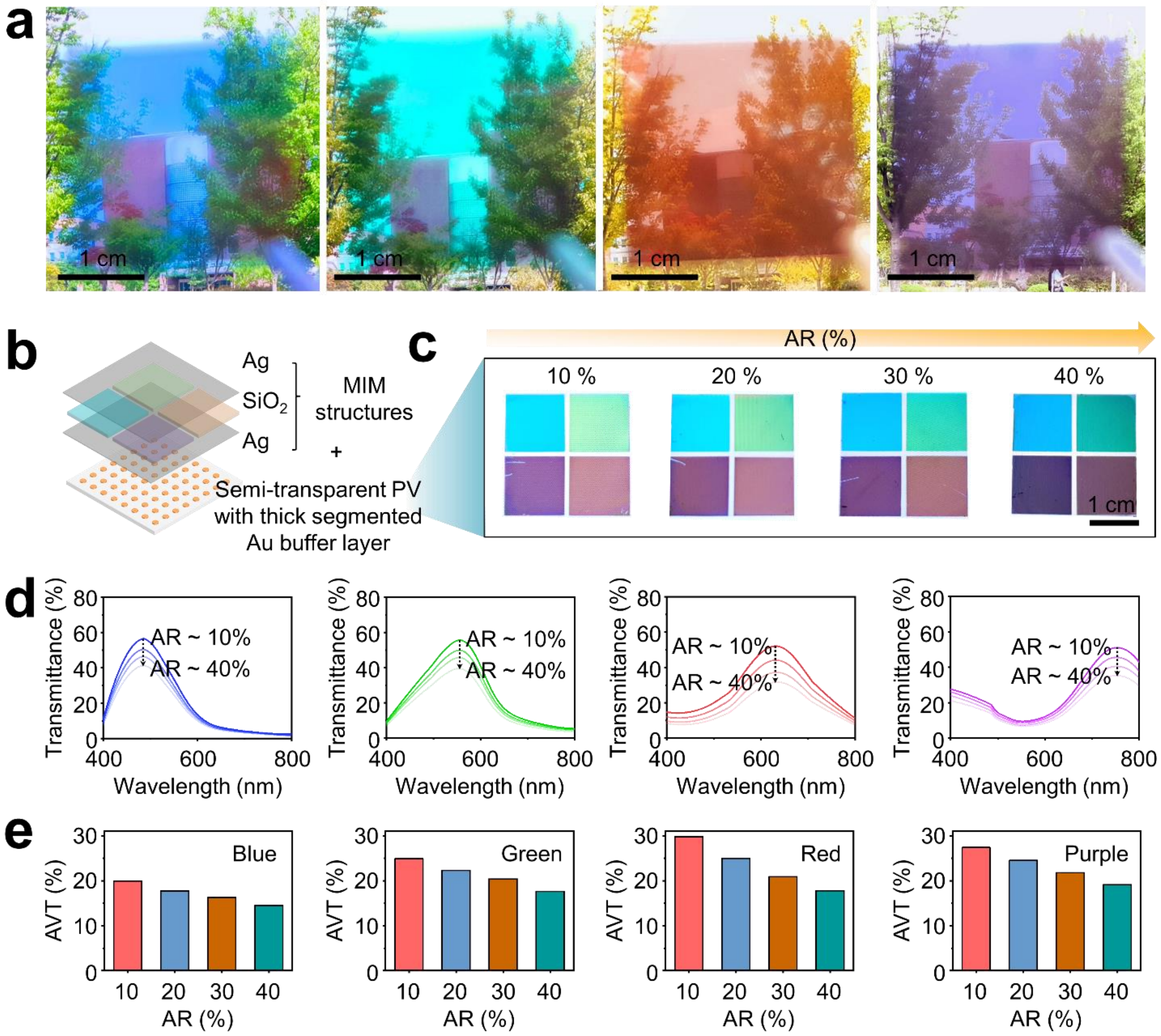

Supplementary Fig. 22 | Color expressions of colored solar windows. a, Outdoor images of four colored solar windows. **b,** Schematic illustration for the design of the colored solar window with four different MIM structures. The MIM structure was added on the backside of the semi-transparent PV with the thick segmented Au buffer layer (70 nm). **c,** Optical camera images of colored solar windows in (b) with various ARs (10–40 %). **d** and **e,** Measurement data of the light transmittance (d) and AVTs (e) for four types of colored solar windows with four different MIM structures that express blue, green, red, and purple.

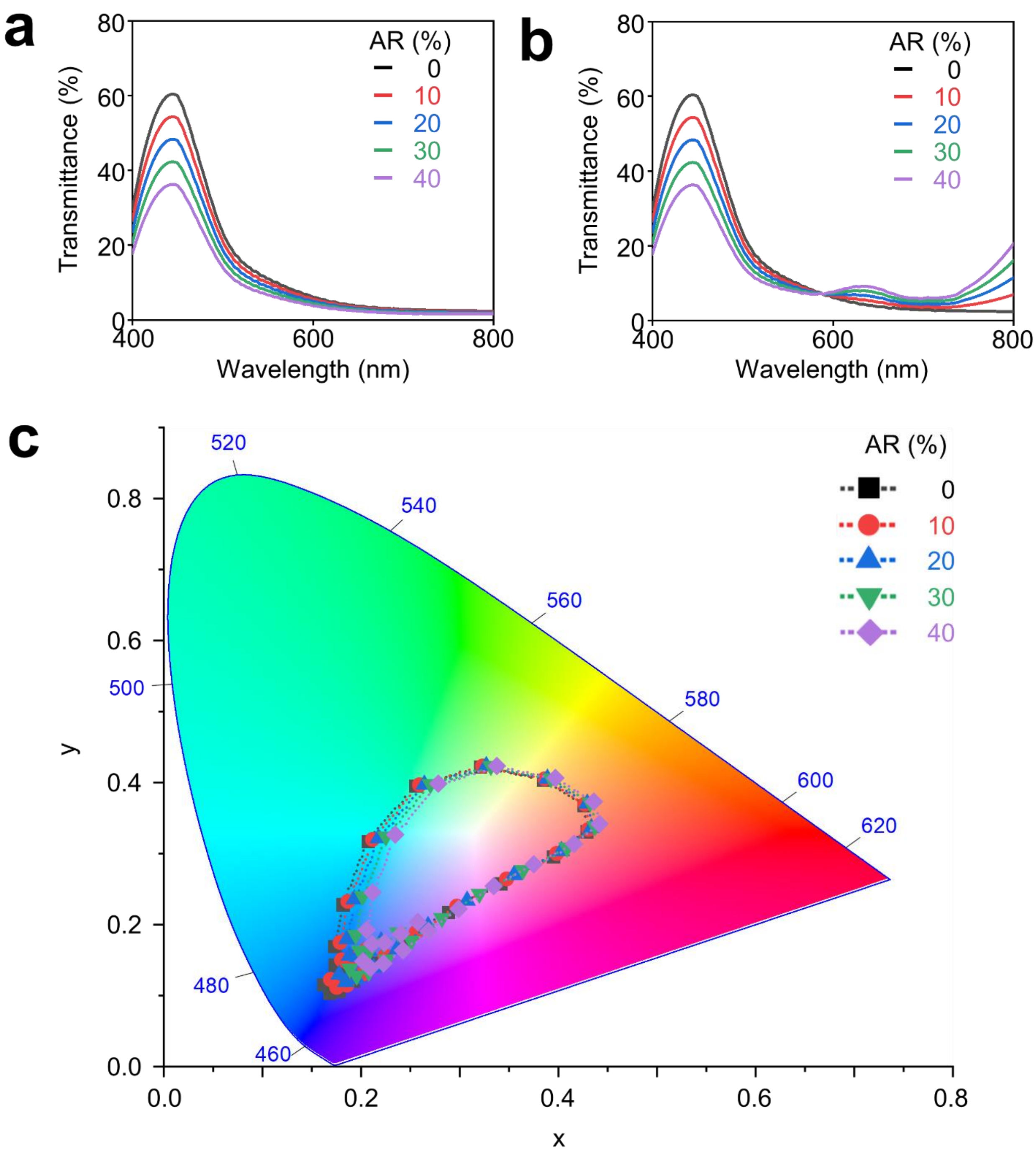

Supplementary Fig. 23 | Color expressions of colored solar windows with two different thicknesses of the Au buffer layer. a, Transmittance spectra of the colored solar windows with various ARs. The thicknesses of the Au buffer layer and insulator are 70 nm and 80 nm, respectively. **b,** Transmittance spectra of the colored solar windows with various ARs. The thickness of the Au buffer layer and insulator are 6 nm and 80 nm, respectively. **c,** CIE coordinates that correspond to the change of t_{ins} between 50 and 250 nm with various ARs. The thickness of the Au buffer layer is 6 nm.

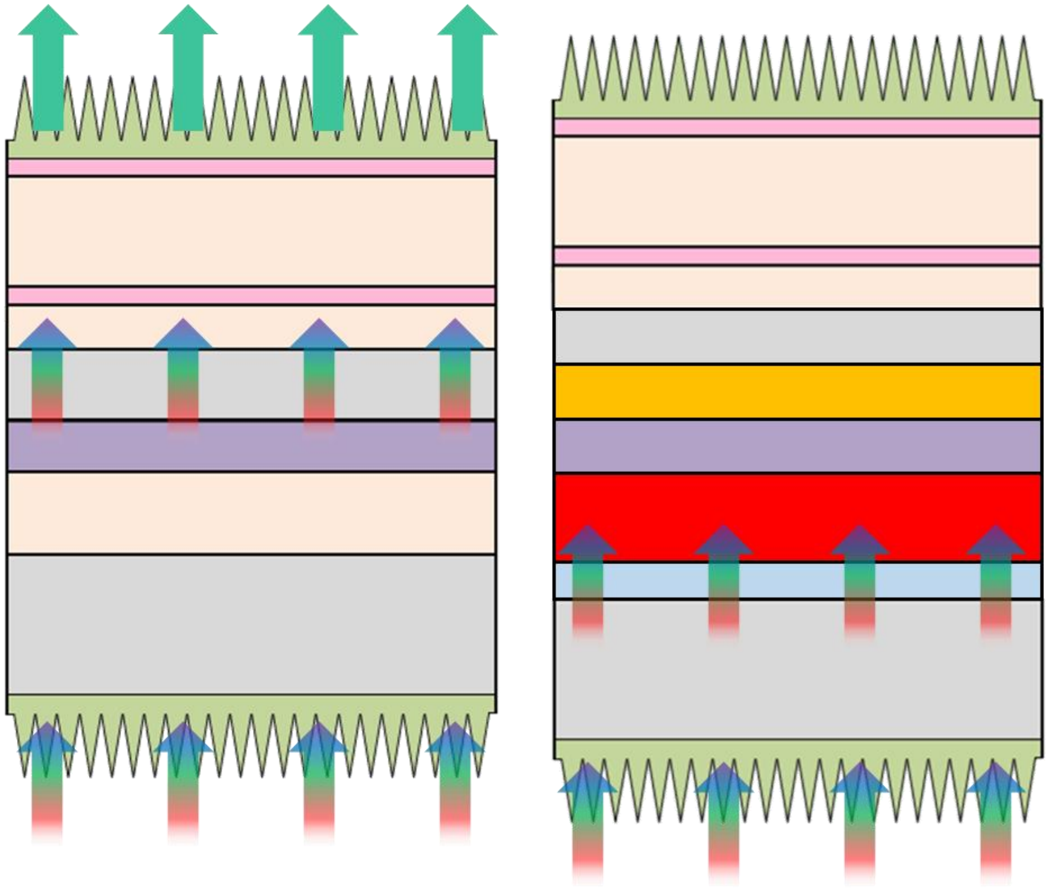

Supplementary Fig. 24 | The structure of the colored solar window with the moth-eye structure. Schematic illustration of the structure of the colored solar window. Light entering from the front side is selectively transmitted through the MIM structure in the non-microcell area (left), while in microcell area, it is absorbed by the perovskite layer (right).

AVT (%)	PCE (%)	LUE (a.u.)	CRI (%)	LUE x CRI (a.u.)	Reference
**72.70**	**2.04**	**1.48**	**98.9**	**147**	**AR ~ 10 % w/o moth-eye**
**64.33**	**3.96**	**2.55**	**97.6**	**249**	**AR ~ 20 % w/o moth-eye**
**56.91**	**5.78**	**3.29**	**97.3**	**320**	**AR ~ 30 % w/o moth-eye**
**50.04**	**7.74**	**3.87**	**97.2**	**376**	**AR ~ 40 % w/o moth-eye**
**42.98**	**9.01**	**3.87**	**99.0**	**383**	**AR ~ 50 % w/o moth-eye**
**35.20**	**11.01**	**3.87**	**98.3**	**381**	**AR ~ 60 % w/o moth-eye**
**56.45**	**8.28**	**4.67**	**97.5**	**455**	**AR ~ 40 % w/ moth-eye**
52.91	10.55	5.58	63.9	357	1
27.5	11.7	3.22	89.5	288	2
63	4.6	2.90	93.9	272	3
37.5	7.53	2.82	59.8	169	4
25.5	10.8	2.75	21.7	60	5
40	6.7	2.68	98.1	263	6
61	4.02	2.45	94.9	233	7
28.4	8.5	2.41	40.6	98	8
31.8	7.47	2.38	68.6	163	9
47.3	5	2.37	97.3	230	10
75.8	3.11	2.36	88.3	208	11
38	6.1	2.32	92.8	215	12
17.3	12.55	2.17	19.8	43	13
47	4.5	2.12	78.8	167	14
24.1	8.4	2.02	76.4	155	15
29	6.41	1.86	40.7	76	16
21.7	8.49	1.84	83.5	154	17
38.62	4.27	1.65	92.4	152	18
31	5.3	1.64	48.5	80	19
23.6	6.92	1.63	24.7	40	20
27.9	5.62	1.57	91.7	144	21
21.6	6.83	1.48	97.4	144	22
25.5	5.27	1.34	75.4	101	23
31.71	4.22	1.34	88.4	118	24
41	2.9	1.19	86.1	102	25
24	4.9	1.18	92.8	109	26
42.1	2.4	1.01	89.5	90	27
60.1	1.57	0.94	82.2	78	28

Supplementary Table 1 | AVTs, PCEs, LUEs, CRIs and LUE × CRIs of semi-transparent PVs. The CRIs, which did not include in the original reference data, were calculated[50].

Area ratio (%)	Scan direction	V_{oc} (V)	J_{sc} (mA/cm^2)	FF	PCE (%)
10 (w/o Moth-eye)	Forward	1.11	2.45	72.1	1.97
	Reverse	1.11	2.48	76.4	2.10
20 (w/o Moth-eye)	Forward	1.12	4.87	69.7	3.81
	Reverse	1.13	4.73	77.2	4.12
30 (w/o Moth-eye)	Forward	1.11	7.16	69.9	5.57
	Reverse	1.12	6.97	76.7	5.98
40 (w/o Moth-eye)	Forward	1.12	9.17	74.8	7.68
	Reverse	1.13	9.11	75.9	7.79
40 (w/o Moth-eye) champion cell	Forward	1.15	10.1	63.2	7.35
	Reverse	1.15	10.2	69.5	8.12
40 (w/ Moth-eye) champion cell	Forward	1.14	10.8	64.7	8.01
	Reverse	1.13	10.7	70.5	8.54

Supplementary Table 2 | Photovoltaic parameters of semi-transparent PVs.

AR (%)	AVT (%)	x of CIE coordination	y of CIE coordination	Haze (%)
**10**	**72.7**	0.3132	0.3338	0.91
**20**	**64.3**	0.3127	0.3306	1.01
**30**	**56.9**	03174	0.3354	1.73
**40**	**50.0**	0.3181	0.3343	2.42
**50**	**43.0**	0.3159	0.3354	2.67
**60**	**35.2**	0.3146	0.3315	2.66

Supplementary Table 3 | Optical properties of semi-transparent PVs.

Avg. Saturation	Avg. AVT (%)	Avg. PCE (%)	Avg. (LUE x Saturation)	Reference
**53.89**	**26.8**	**8.09**	**116.8**	**AR ~ 40 % w/ moth-eye**
67.50	6.18	3.46	14.4	51
96.52	3.73	6.17	22.2	52
96.25	3.97	10.77	41.2	53
98.83	5.24	5	25.9	54
67.22	9.2	11.15	69.0	55
86.41	5.92	13.43	68.7	55
96.97	1.05	15.59	15.9	55
77.74	10.24	3.43	27.3	56
82.64	8.2	5.58	37.8	57
80.74	10.87	4.5	39.5	57
80.08	9.54	4.86	37.1	57
87.32	13.47	4.44	52.2	58

Supplementary Table 4 | Saturation, AVTs, PCEs, and LUE × Saturation of the colored PVs. The values are averaged using each value of red, blue, and green (RGB) colored PVs[51-58].

Supplementary References

1. Zuo. L., Shi, X., Fu, W., & Jen, A. K.-Y. Highly efficient semitransparent solar cells with selective absorption and tandem architecture. *Adv. Mater.* **31**, 1901683 (2019).
2. Chen, B.-X. *et al.* Ordered macroporous CH3NH3PbI3 perovskite semitransparent film for high-performance solar cells. *J. Mater. Chem. A* **4**, 15662 (2016).
3. Chen, C.-C. *et al.* High-performance semi-transparent polymer solar cells possessing tandem structures. *Energy Environ. Sci.* **6**, 2714 (2013).
4. Jung, J. W., Chueh, C.-C. & Jen, A. K.-Y. High-performance semitransparent perovskite solar cells with 10% power conversion efficiency and 25% average visible transmittance based on transparent CuSCN as the hole-transporting material. *Adv. Energy Mater.* **5**, 1500486 (2015).
5. Chang, C.-Y. *et al.* High-performance, air-Stable, low-temperature processed semitransparent perovskite solar cells enabled by atomic layer deposition. *Chem. Mater.* **27**, 5122-5130 (2015).
6. Chang, C.-Y. *et al.* Highly efficient polymer tandem cells and semitransparent cells for solar energy. *Adv. Energy Mater.* **4**, 1301645 (2014).
7. Chen, C.-C. *et al.* Visibly transparent polymer solar cells produced by solution processing. *ACS Nano* **6**, 7185-7190 (2012).
8. Guo, F. *et al.* High-performance semitransparent perovskite solar cells with solution-processed silver nanowires as top electrodes. *Nanoscale* **7**, 1642-1649 (2015).
9. Cui, Y. *et al.* Efficient semitransparent organic solar cells with tunable color enabled by an ultralow-bandgap nonfullerene acceptor. *Adv. Mater.* **29**, 1703080 (2017).
10. Chang, C.-Y. *et al.* A versatile fluoro-containing low-bandgap polymer for efficient semitransparent and tandem polymer solar cells. *Adv. Funct. Mater.* **23**, 5084-5090 (2013).
11. Yang, C. *et al.* Ultraviolet and near-infrared dual-band selective-harvesting transparent luminescent solar concentrators. *Adv. Energy Mater.* **11**, 2003581 (2021).
12. Horantner, M. T. *et al.* Shunt-blocking layers for semitransparent perovskite solar cells. *Adv. Mater. Interfaces* **3**, 1500837 (2016).
13. Heo, J. H. et al. Stable semi-transparent CH3NH3PbI3 planar sandwich solar cells. *Energy Environ. Sci.* **8**, 2922-2927 (2015).
14. Bag, S. & Durstock, M. F. Efficient semi-transparent planar perovskite solar cells using a 'molecular glue'. *Nano Energy* **30**, 542-548 (2016).
15. Dastjerdi, H. T., Qi, P., Fan, Z. & Tavakoli, M. M. Cost-effective and semi-transparent PbS quantum dot solar cells using copper electrodes. *ACS Appl. Mater. Interfaces* **12**, 818-825 (2020).
16. Quiroz, C. O. R. *et al.* Pushing efficiency limits for semitransparent perovskite solar cells. *J. Mater. Chem. A* **3**, 24071-24081 (2015).
17. Li, F. R. *et al.* Nanotube enhanced carbon grids as top electrodes for fully printable mesoscopic semitransparent perovskite solar cells. *J. Mater. Chem. A* **5**, 10374-10379 (2017).
18. Zhang, Y. et al. Colorful semitransparent polymer solar cells employing a bottom periodic one-dimensional photonic crystal and a top conductive PEDOT:PSS layer. *J. Mater. Chem. A* **4**, 11821 (2016).
19. Gaspera, E. D. *et al.* Ultra-thin high efficiency semitransparent perovskite solar cells. *Nano Energy* **13**, 249-257 (2015).

20. Lim, J. W. *et al.* Highly transparent amorphous silicon solar cells fabricated using thin absorber and high-bandgap-energy n/i-interface layers. *Solar Energy Materials & Solar cells* **128**, 301-306 (2014).
21. Chen, K.-S. *et al.* Semi-transparent polymer solar cells with 6% pce, 25% average visible transmittance and a color rendering index close to 100 for power generating window applications. *Energy Environ. Sci.* **5**, 9551 (2012).
22. Yin, Z. *et al.* Long lifetime stable and efficient semitransparent organic solar cells using a ZnMgO-modified cathode combined with a thin MoO3/Ag anode. *J. Mater. Chem. A* **5**, 3888 (2017).
23. Saifullah, M. *et al*. Development of semitransparent CIGS thin-film solar cells modified with a sulfurized-AgGa layer for building applications. *J. Mater. Chem. A*. **4**, 10542-10551 (2016).
24. Chueh, C.-C. *et al.* Toward High-Performance Semi-transparent polymer solar cells: optimization of ultra-thin light absorbing layer and transparent cathode architecture. *Adv. Energy Mater.* **3**, 417-423 (2013).
25. Guo, F. *et al.* Fully solution-processing route toward highly transparent polymer solar cells. *ACS Appl. Mater. Interfaces* **6**, 18251-18257 (2014).
26. Meiss, J. *et al.* Highly efficient semitransparent tandem organic solar cells with complementary absorber materials. *Appl. Phys. Lett.* **99**, 043301 (2011).
27. Meiss, J. *et al.* Near-infrared absorbing semitransparent organic solar cells. *Appl. Phys. Lett.* **99**, 193307 (2011).
28. Sumner, R. *et al.* Analysis of optical losses in high-efficiency CuInS2-based nanocrystal luminescent solar concentrators: balancing absorption versus scattering. *J. Phys. Chem. C* **121**, 3252-3260 (2017).
29. Yang, C. *et al.* High-performance near-infrared harvesting transparent luminescent solar concentrators. *Adv. Optical. Mater.* **8**, 1901536 (2020).
30. Zhang, B. *et al.* High-performance large-area luminescence solar concentrator incorporating a donor− emitter fluorophore system. *ACS Energy Lett.* **4**, 1839-1844 (2019).
31. Lunt, R. R. & Bulovic, V. Transparent, near-infrared organic photovoltaic solar cells for window and energy-scavenging applications. *Appl. Phys. Lett.* **98**, 113305 (2011).
32. Liu, D. *et al.* Lead halide ultraviolet-harvesting transparent photovoltaics with an efficiency exceeding 1%. *ACS. Appl. Energy Mater*. **2**, 3972-3978 (2019).
33. Bergren, M. R. *et al.* High-performance CuInS2 quantum dot laminated glass luminescent solar concentrators for windows. *ACS Energy Lett.* **3**, 520-525 (2018).
34. Bu, L. *et al.* Semitransparent fully air processed perovskite solar cells. *ACS Appl. Mater. Interfaces* **7**, 17776-17781 (2015).
35. Wong, Y. Q. *et al.* Efficient semitransparent organic solar cells with good color perception and good color rendering by blade coating. *Org. Electron.* **43**, 196-206 (2017).
36. Zhou, Y. et al. Near infrared, highly efficient luminescent solar concentrators. *Adv. Energy Mater.* **6**, 1501913 (2016).
37. Brzeczek-Szafran, A. *et al.* Aesthetically pleasing, visible light transmissive, luminescent solar concentrators using a BODIPY derivative. *Phys. Status Solidi* **215**, 1800551 (2018).
38. Meinardi, F. *et al.* Highly efficient luminescent solar concentrators based on earth-abundant indirect-bandgap silicon quantum dots. *Nat. Photonics* **11**, 177-185 (2017).
39. Yang, C. *et al.* Impact of stokes shift on the performance of near-infrared harvesting transparent luminescent solar concentrators. *Sci. Rep.* **8**, 16359 (2018).

40. Li, H. *et al.* Doctor-blade deposition of quantum dots onto standard window glass for low-loss large-area luminescent solar concentrators. *Nat. Energy* **1**, 16157 (2016).
41. Meinardi, F. *et al.* Highly efficient large-area colourless luminescent solar concentrators using heavy-metal-free colloidal quantum dots. *Nat. Nanotech.* **10**, 878-885 (2015).
42. Liu, D., Yang, C. & Lunt, R. R. Halide perovskites for selective ultraviolet-harvesting transparent photovoltaics. *Joule* **2**, 1827-1837 (2018).
43. Zhao, Y. & Lunt, R. R. Transparent luminescent solar concentrators for large-area solar windows enabled by massive stokes-shift nanocluster phosphors. *Adv. Energy Mater.* **3**, 1143-1148 (2013).
44. Rondao, R. *et al.* High-performance near-infrared luminescent solar concentrators. *ACS Appl. Mater. Interfaces* **9**, 12540-12546 (2017).
45. Czolk, J. *et al.* Highly efficient, mechanically flexible, semi-transparent organic solar cells doctor bladed from non-halogenated solvents. *Adv. Mater. Technol.* **1**, 1600184 (2016).
46. Zhao, Y. et al. Near-infrared harvesting transparent luminescent solar concentrators. *Adv. Optical Mater.* **2**, 606-611 (2014).
47. Brennan, L. J. et al. Large area quantum dot luminescent solar concentrators for use with dye-sensitised solar cells. *J. Mater. Chem. A* **6**, 2671 (2018).
48. Lee, J.-Y., Connor, S. T., Cui, Y. & Peumans, P. Semitransparent organic photovoltaic cells with laminated top electrode. *Nano Lett*. **10**, 1276-1279 (2010).
49. Krumer, Z. *et al.* Compensation of self-absorption losses in luminescent solar concentrators by increasing luminophore concentration. *Solar Energy Materials and Solar Cells* **167**, 133-139 (2017).
50. Yang, C., Liu, D. & Lunt, R. R. How to accurately report transparent luminescent solar concentrators. *Joule* **3**, 1-6 (2019).
51. Lee, K.-T., Fukuda, M., Joglekar, S. & Guo, L. J. Colored, see-through perovskite solar cells employing an optical cavity. *J. Mater. Chem. C* **3**, 5377-5382 (2015).
52. Lu, J.-H. *et al.* High-performance, semitransparent, easily tunable vivid colorful perovskite photovoltaics featuring Ag/ITO/Ag microcavity structures. *J. Phys. Chem. C* **120**, 4233-4239 (2016).
53. Lee, K.-T. *et al.* High-performance colorful semitransparent perovskite solar cells with phase-compensated microcavities. *Nano Research* **11**, 2553-2561 (2018).
54. Chen, Y.-H. *et al.* Microcavity-embedded, colour-tuneable, transparent organic solar cells. *Adv. Mater.* **26**, 1129-1134 (2014).
55. Li, X. *et al.* Semitransparent organic solar cells with vivid colors. *ACS Energy Lett.* **5**, 3115-3123 (2020).
56. Quiroz, C. O. R. *et al.* Coloring semitransparent perovskite solar cells via dielectric mirrors. *ACS Nano* **10**, 5104-5112 (2016).
57. Shafian, S. *et al.* Active-material-independent color-tunable semitransparent organic solar cells. *ACS Appl. Mater. Interfaces* **11**, 18887-18895 (2019).
58. Kim, Y. *et al.* Semitransparent blue, green, and red organic solar cells using color filtering electrodes. *Adv. Optical Mater.* **6**, 1800051 (2018).
59. Lee, W. *et al*. High-resolution spin-on-patterning of perovskite thin films for a multiplexed image sensor array. *Adv. Mater*. **29**, 1702902 (2017).
60. Coburn, J. W. & Winters, H. F. Ion- and electron-assisted gas-surface chemistry—An important effect in plasma etching. *J. Appl. Phys.* **50**, 3189-3196 (1979).

61. Bett, A. J. *et al*. Semi-transparent perovskite solar cells with ITO directly sputtered on Spiro-OMeTAD for tandem applications. *ACS Appl. Mater. Interfaces* **11**, 45796-45804 (2019).
