# Supplementary material for: Perovskite microcells fabricated using swelling-induced crack propagation for colored solar windows
Source: Nat Commun. 2022 Apr 11;13:1946. doi: 10.1038/s41467-022-29602-z (PMC9001655; doi:10.1038/s41467-022-29602-z)
Supplement: Supplementary file 2 — Description of Additional Supplementary Files [file 41467_2022_29602_MOESM2_ESM.docx]

**Description of Additional Supplementary Files**

**File Name: Supplementary Movie 1
Description:** Peeling-off procedure of the PMMA/PI/perovskite multi-layers.
