# Supplementary material for: Perovskite microcells fabricated using swelling-induced crack propagation for colored solar windows
Source: Nat Commun. 2022 Apr 11;13:1946. doi: 10.1038/s41467-022-29602-z (PMC9001655; doi:10.1038/s41467-022-29602-z)
Supplement: Supplementary file 4 — Solar Cells Reporting Summary [file 41467_2022_29602_MOESM4_ESM.pdf]

nature research

Corresponding author(s): Dae-Hyeong Kim
Young Min Song
Dong Hoe Kim

Solar Cells Reporting Summary

Nature Research wishes to improve the reproducibility of the work that we publish. This form is intended for publication with all accepted papers reporting the characterization of photovoltaic devices and provides structure for consistency and transparency in reporting. Some list items might not apply to an individual manuscript, but all fields must be completed for clarity.

For further information on Nature Research policies, including our data availability policy, see Authors & Referees.

ü Experimental design

Please check: are the following details reported in the manuscript?

1. Dimensions

Item	Yes/No	Details
Area of the tested solar cells	[x] Yes [ ] No	An electrode of 9 mm2 was deposited, and microcell area was measured and calibrated by SEM images.
Method used to determine the device area	[x] Yes [ ] No	All details about the device area are added in the methods section.

2. Current-voltage characterization

Item	Yes/No	Details
Current density-voltage (J-V) plots in both forward and backward direction	[x] Yes [ ] No	For microcells, J-V characteristics were plotted in Fig. 3h. For semi-transparent PV, parameters of J-V characteristics were written in Supplementary Table 2.
Voltage scan conditions<br>*For instance: scan direction, speed, dwell times*	[x] Yes [ ] No	All details about the measurement conditions are added in the methods section.
Test environment<br>*For instance: characterization temperature, in air or in glove box*	[x] Yes [ ] No	All details about the measurement conditions are added in the methods section.
Protocol for preconditioning of the device before its characterization	[x] Yes [ ] No	There was no preconditioning to measure the device.
Stability of the J-V characteristic<br>*Verified with time evolution of the maximum power point or with the photocurrent at maximum power point; see ref. 7 for details.*	[x] Yes [ ] No	Given in Fig. 3i.

3. Hysteresis or any other unusual behaviour

Item	Yes/No	Details
Description of the unusual behaviour observed during the characterization	[x] Yes [ ] No	The poor quality of the perovskite film fabricated by the dewetting method with a conventional structure led to poor device characteristics including hysteresis (Fig. 3h).
Related experimental data	[x] Yes [ ] No	Given in Fig. 3h

4. Efficiency

Item	Yes/No	Details
External quantum efficiency (EQE) or incident photons to current efficiency (IPCE)	[x] Yes [ ] No	EQE was measured for perovskite microcell with an area 1.5mm x 1.5mm. However, we could not obtain reliable EQE data from semi-transparent and colored PVs since the spot size (0.8 mm x 1.0mm) of EQE measurement system is much larger than the diameter of microcells that make up the semi-transparent and colored PVs (~100um)
A comparison between the integrated response under the standard reference spectrum and the response measure under the simulator	[x] Yes [ ] No	The integrated Jsc calculated from EQE is 21.76 mA/cm2, which is well matched (within 4 %) to the measured Jsc under the solar simulator.
For tandem solar cells, the bias illumination and bias voltage used for each subcell	[ ] Yes [x] No	Our PVs are not tandem solar cells.

5. Calibration

Item	Yes/No	Details
Light source and reference cell or sensor used for the characterization	[x] Yes [ ] No	Solar simulator was calibrated using the reference cell purchased from Newport.

Confirmation that the reference cell was calibrated and certified

- [x] Yes
- [ ] No

Our reference cell was calibrated and certified by Newport.

Calculation of spectral mismatch between the reference cell and the devices under test

- [x] Yes
- [ ] No

The light spectrum used for the measurements well matched the reference cell and AM1.5G illumination, and we used a mismatched factor of 1
for all devices.

6. Mask/aperture

Size of the mask/aperture used during testing

- [ ] Yes
- [x] No

We didn't use the mask for measuring JV characteristics because the cell
area of each pixel was very small. However, the microcell area was measured
and calibrated by SEM images.

Variation of the measured short-circuit current density with the mask/aperture area

- [ ] Yes
- [x] No

We didn't use the mask for the measurment, but PCEs depending on the
diameter of transparent PVs' microcells were measured in Fig. 4c.

7. Performance certification

Identity of the independent certification laboratory that confirmed the photovoltaic performance

- [ ] Yes
- [x] No

Certification is not conducted in this work.

A copy of any certificate(s)
Provide in Supplementary Information

- [ ] Yes
- [x] No

Certification is not conducted in this work.

8. Statistics

Number of solar cells tested

- [x] Yes
- [ ] No

25 PVs were tested (Fig. 3k).

Statistical analysis of the device performance

- [x] Yes
- [ ] No

Given in Fig. 3k.

9. Long-term stability analysis

Type of analysis, bias conditions and environmental conditions
For instance: illumination type, temperature, atmosphere humidity, encapsulation method, preconditioning temperature

- [ ] Yes
- [x] No

Long-term stability analysis is not conducted in this work.
